# ClbR Is the Key Transcriptional Activator of Colibactin Gene Expression in Escherichia coli

**DOI:** 10.1128/mSphere.00591-20

**Published:** 2020-07-15

**Authors:** Alexander Wallenstein, Nadine Rehm, Marina Brinkmann, Martina Selle, Nadège Bossuet-Greif, Daniel Sauer, Boyke Bunk, Cathrin Spröer, Haleluya Tesfaye Wami, Stefan Homburg, Rudolf von Bünau, Simone König, Jean-Philippe Nougayrède, Jörg Overmann, Eric Oswald, Rolf Müller, Ulrich Dobrindt

**Affiliations:** a Institute of Hygiene, University of Münster, Münster, Germany; b Interdisciplinary Center for Clinical Research, Medical Faculty, University of Münster, Münster, Germany; c Institute of Molecular Infection Biology, University of Würzburg, Würzburg, Germany; d IRSD, Université de Toulouse, INSERM, INRA, ENVT, UPS, Toulouse, France; e Department of Microbial Natural Products, Helmholtz Institute for Pharmaceutical Research Saarland, Helmholtz Center for Infection Research, Saarland University, Saarbrücken, Germany; f Leibniz Institute DSMZ—German Collection of Microorganisms and Cell Cultures, Braunschweig, Germany; g DZIF—German Center for Infection Research, Hannover-Braunschweig Partner Site, Braunschweig, Germany; h Pharma Zentrale GmbH, Herdecke, Germany; i Core Unit Proteomics, Interdisciplinary Center for Clinical Research, Medical Faculty, University of Münster, Münster, Germany; University of Iowa

**Keywords:** secondary metabolite, polyketide, cytopathic effect, RNA-seq, VNTR

## Abstract

Colibactin is a nonribosomal peptide/polyketide hybrid natural product expressed by different members of the *Enterobacteriaceae* which can be correlated with induction of DNA double-strand breaks and interference with cell cycle progression in eukaryotes. Regulatory features of colibactin expression are only incompletely understood. We used Escherichia coli strain M1/5 as a model to investigate regulation of expression of the colibactin determinant at the transcriptional level and to characterize regulatory elements located within the colibactin pathogenicity island itself. We measured *clbR* transcription *in vitro* and observed that cultivation in defined minimal media led to increased colibactin expression relative to rich media. Transcription of *clbR* directly responds to iron availability. We also characterized structural DNA elements inside the colibactin determinant involved in ClbR-dependent regulation, i.e., ClbR binding sites and a variable number of tandem repeats located upstream of *clbR*. We investigated the impact of *clbR* overexpression or deletion at the transcriptome and proteome levels. Moreover, we compared global gene regulation under these conditions with that occurring upon overexpression or deletion of *clbQ*, which affects the flux of colibactin production. Combining the results of the transcriptome and proteome analyses with indirect measurements of colibactin levels by cell culture assays and an approximate quantification of colibactin via the second product of colibactin cleavage from precolibactin, N-myristoyl-d-asparagine, we demonstrate that the variable number of tandem repeats plays a significant regulatory role in colibactin expression. We identify ClbR as the only transcriptional activator known so far that is specific and essential for efficient regulation of colibactin production.

**IMPORTANCE** The nonribosomal peptide/polyketide hybrid colibactin can be considered a bacterial virulence factor involved in extraintestinal infection and also a procarcinogen. Nevertheless, and despite its genotoxic effect, colibactin expression can also inhibit bacterial or tumor growth and correlates with probiotic anti-inflammatory and analgesic properties. Although the biological function of this natural compound has been studied extensively, our understanding of the regulation of colibactin expression is still far from complete. We investigated in detail the role of regulatory elements involved in colibactin expression and in the growth conditions that promote colibactin expression. In this way, our data shed light on the regulatory mechanisms involved in colibactin expression and may support the expression and purification of this interesting nonribosomal peptide/polyketide hybrid for further molecular characterization.

## Abstract (precis)

The nonribosomal peptide/polyketide hybrid colibactin can be considered a bacterial virulence factor involved in extraintestinal infection and also a procarcinogen. Nevertheless, and despite its genotoxic effect, colibactin expression can also inhibit bacterial or tumor growth and correlates with probiotic anti-inflammatory and analgesic properties. Although the biological function of this natural compound has been studied extensively, our understanding of the regulation of colibactin expression is still far from complete. We investigated in detail the role of regulatory elements involved in colibactin expression and in the growth conditions that promote colibactin expression. In this way, our data shed light on the regulatory mechanisms involved in colibactin expression and may support the expression and purification of this interesting nonribosomal peptide/polyketide hybrid for further molecular characterization.

## INTRODUCTION

Certain members of the family of *Enterobacteriaceae* are able to produce the hybrid nonribosomal peptide/polyketide natural product colibactin (1, 2). The ability to express this cyclomodulin has so far been described in strains of Escherichia coli, Citrobacter koseri, Klebsiella pneumoniae, and Klebsiella aerogenes (formerly known as Enterobacter aerogenes). Many colibactin-positive isolates are pathogenic, but commensal fecal strains can express this compound as well, and even certain probiotic traits have been correlated with the presence of the so-called *pks* island harboring the colibactin determinant (3, 4). This 54-kb island comprises 19 genes, encoding products required for the biosynthesis and transport of functional colibactin (2). Colibactin has been shown to be a virulence factor (VF) of extraintestinal pathogenic E. coli (ExPEC) strains (5–7), but, due to its ability to cause DNA double-strand breaks, DNA cross-links, and chromosome instability (8–11) together with its presence in E. coli strains isolated from biopsy specimens of colorectal cancer patients (12, 13), it is also discussed as a procarcinogen (14). At the same time, colibactin expression was reported to inhibit bacterial or tumor growth (10, 15). An alternative function as a bacteriocin has also been discussed (16). Experimental evidence has been provided indicating that colibactin expression is linked to probiotic anti-inflammatory (4) and analgesic (17) activities of E. coli Nissle 1917 (EcN). Furthermore, components of the colibactin biosynthesis machinery are also involved in microcin M and H47 biosynthesis (3). Extensive efforts have been invested into the elucidation of the biosynthesis pathway, structure, and mode of action of this secondary metabolite (11, 18–20) in order to understand its biological function (21).

Colibactin biosynthesis by the enzymatic assembly line starts with activation of the nonribosomal peptide synthetases and polyketide synthases by the phosphopantetheinyl transferase ClbA. The first building block channeled into colibactin synthesis is an asparagine, which is processed first by ClbN, followed by ClbB (22), and then the biosynthesis continues with the action of the proteins ClbC-H-I-J-K, incorporating also an aminomalonyl unit generated by the enzymes ClbD-E-F-G (23–25). This intermediate is completed by the action of ClbO-L and then undergoes an editing process mediated by the atypical thioesterase ClbQ (26, 27). It is then transported to the periplasm by the activity of the multidrug and toxic compound extrusion (MATE) transporter ClbM (28), where the precolibactin is finally matured by the peptidase activity of ClbP (29). Colibactin-producing bacteria protect themselves against the DNA damaging activity of this compound by expressing ClbS, a resistance protein, which binds and deactivates colibactin (30). Whether colibactin is subsequently presented on the bacterial cell surface or is released into the medium or actively secreted into host cells remains unclear. In general, the processes involved in the uptake of colibactin in host cells are still largely unknown. Direct bacterium-host cell contact is necessary for internalization of colibactin into host cells, which is limited by the presence of an intact cell membrane or mucus layer (31, 32). Once colibactin is internalized, its ability to cross-link DNA lays the foundation for its cell cycle modifying effect via the induction of DNA double-strand breaks (8). How colibactin finds its way into the nucleus has not been described thus far.

**FIG 1:**
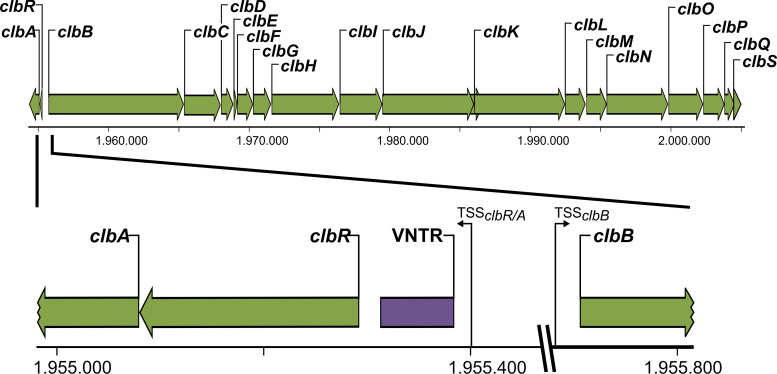
Genetic structure of the colibactin determinant in E. coli strains of phylogenetic group B2. The 54-kb colibactin island consists of two units. The smaller part is necessary for the activation of colibactin genes and for expression of genes encoding enzymes involved in colibactin production, including *clbA* and *clbR*, encoding a posphopantetheinyltransferase and a transcriptional activator, respectively. The larger part of the determinant, located on the opposing strand, contains genes *clbB* to *clbS* coding for components required for biosynthesis, transport, and resistance against colibactin. The intergenic region between *clbR* and *clbB* comprises regions with variable numbers of tandem repeats (VNTR), which in the case of E. coli strain M1/5 consists of nine repeats of the octanucleotide sequence ACAGATAC. The intergenic region and its flanking sequence context have been enlarged.

The genetic organization of the *pks* island exhibits at first sight two distinct features as follows. With the exception of the *clbR* and *clbA* genes, all of the *clb* genes are organized in the same orientation with no or only short (∼50-bp) intergenic regions. The gene cluster coding for the components of the colibactin assembly line starts with *clbB* and ends with the resistance gene *clbS* (Fig. 1). Several of these genes are polycistronically transcribed (1, 33). Another smaller but no less important gene cluster is oriented in the opposite direction, is separated by an approximately 400-bp intergenic region from *clbB*, and codes for the phosphopantetheinyl transferase ClbA and the designated transcriptional activator ClbR (1, 33). Both gene products are necessary for the activation of colibactin production, either by switching on the synthesis proteins or by regulation of colibactin gene transcription. Upstream of these initiating genes, a specific structural element is located, i.e., a region with variable numbers of tandem repeats (VNTR) (Fig. 1). This region consists of an 8-bp nucleotide sequence, 5′-ACAGATAC-3′, and can vary in size, with 2 to 20 repeats of the octanucleotide sequence, depending on the individual bacterial isolate (2). So far, only the numbers of repeats present in the VNTR region have been described to differ between different strains (2), but it is yet not clear whether variations in the size of the VNTR region affect colibactin expression. On the basis of the localization in the intergenic region between the regulatory gene cluster (*clbR*-*A*) and the biosynthesis gene cluster (*clbB*-*S*), we hypothesize that this genetic element may affect regulation of colibactin expression.

Most of the previous research on colibactin focused on elucidation of the molecular structure and of the mode of action of the active compound or functional intermediates thereof. In contrast, the aim of the present work was to achieve a better understanding of the mechanisms of regulation of colibactin expression and their implications for the biological role of colibactin. The observation that the colibactin biosynthetic pathway can produce different small compounds, such as an analgesic peptide, which can have completely different effects than colibactin itself (14, 15, 17, 22), serves as a great motivator to advance the elucidation of the factors and processes that contribute to the regulation of colibactin expression. Our understanding of the regulation of *pks* island expression revolves around the activity of its proposed key regulator, ClbR. Until now, ClbR has been described as a LuxR-like protein with a helix-turn-helix DNA-binding motif (33), suggesting that this protein is involved in regulation of *pks* island transcription. Therefore, our aim in this study was to characterize the function of ClbR as a transcriptional regulator and further details of the regulation of colibactin expression in E. coli.

(Data reported in this study appeared in part in the diploma thesis of M. Selle, the M.Sc. thesis of M. Brinkmann, and the Ph.D. thesis of A. Wallenstein.)

## RESULTS

### Genome sequence analysis of fecal E. coli isolate M1/5.

For the detailed investigation of regulatory aspects of colibactin expression, we selected E. coli strain M1/5 as our main model organism. This fecal isolate from a healthy volunteer is a reliable colibactin producer and represents the highly relevant group of colibactin-positive E. coli strains of phylogroup B2, which colonize the intestinal tract of many humans. On the basis of the complete genome sequence, E. coli M1/5 was allocated to sequence type 550 (ST550)/clonal complex 14 (CC14) and serotype O75:K5:H5. In addition to the 5,138,587-bp chromosome, the M1/5 genome includes two plasmids, pM1/5-120 (119,964 bp) and pM1/5-30 (29,585 bp) (see Fig. S1 in the supplemental material). Although E. coli M1/5 is a colibactin-positive strain, its genome does not contain many E. coli virulence-associated genes such as those encoding characteristic toxins and adhesins of intestinal or extraintestinal pathogenic E. coli. A group II capsule (serotype K5) gene, multiple fimbrial adhesin operons, several autotransporter-encoding genes, two type six secretion system genes, and different siderophore system genes may contribute to the fitness and competitiveness of E. coli M1/5. Further characteristics regarding the E. coli M1/5 genome content are provided in Table S1 in the supplemental material. Determinants coding for common antibiotic resistance phenotypes in E. coli has been detected in the genome sequence of strain M1/5, which is sensitive to colistin, β-lactams, aminoglycosides, sulfonamide/trimethoprim, phenicols, glycopeptides, tetracyclines, quinolones, rifampin, nitroimidazole, and macrolides.

10.1128/mSphere.00591-20.2 FIG S1 Genetic structure of the two plasmids of E. coli strain M1/5. pM1/5_120 includes a second aerobactin and a Sit siderophore system determinant. The plasmid maps were generated with DNAplotter (87). Plasmid pM1/5-120 (IncF allelic profile F4:A-:B52) displays the greatest partial sequence similarity, over 54 kb, to pYSP8-1-CTX-M-14 (GenBank accession no. CP037912), which was isolated from porcine fecal E. coli isolate YSP8-1, and to an unnamed plasmid from enterotoxigenic E. coli O181:H21 strain D181 (GenBank accession no. CP024249). Additional fitness-related gene clusters present on pM1/5-120 comprise a second copy of the aerobactin iron uptake determinant gene cluster (*iutA*-*iucDCBA*) and the *sitABCD* gene cluster coding for an iron ABC transporter. Plasmid pM1/5-30 could not be allocated to a plasmid incompatibility group but shows the greatest nucleotide sequence similarity to plasmid pEA3 of Erwinia amylovora strain CFBP 2585 (GenBank accession no. HF560646) and pKUN4843_2 of K. pneumoniae isolate KUN4843 (GenBank accession no. LC155909). Although pYSP8-1-CTX-M-14 and pKUN4843_2 encode antibiotic resistance traits, none of the determinants coding for common antibiotic resistance phenotypes in E. coli have been detected in the genome sequence of strain M1/5. Download FIG S1, PDF file, 0.2 MB. Copyright © 2020 Wallenstein et al. 2020 Wallenstein et al. This content is distributed under the terms of the Creative Commons Attribution 4.0 International license.

10.1128/mSphere.00591-20.8 TABLE S1 Fitness-related determinants of fecal isolate E. coli M1/5. Download Table S1, PDF file, 0.04 MB. Copyright © 2020 Wallenstein et al. 2020 Wallenstein et al. This content is distributed under the terms of the Creative Commons Attribution 4.0 International license.

### ClbR is a transcriptional activator of colibactin gene expression.

**FIG 2:**
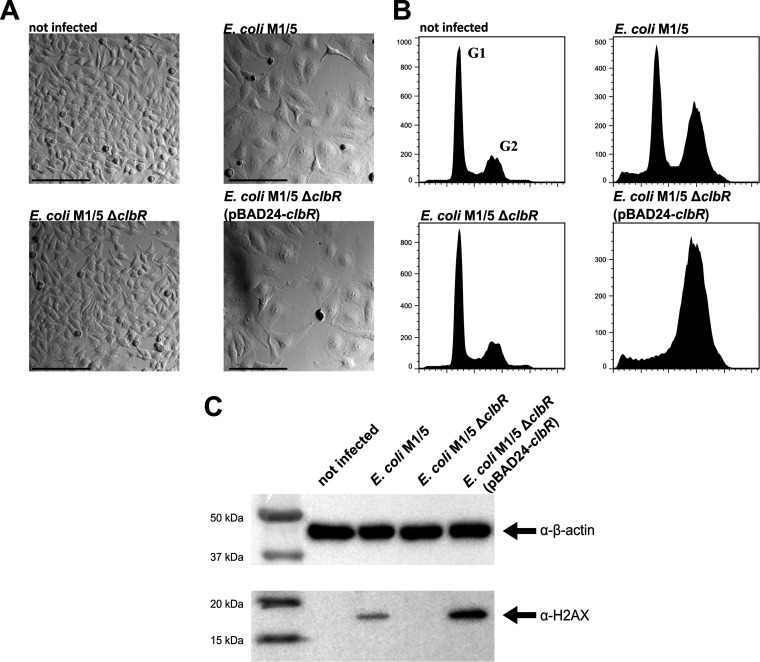
ClbR is a regulator of colibactin expression. (A) HeLa cells were either infected with E. coli strain M1/5 *rpsL*K42R and derivatives (multiplicity of infection [MOI] of 100) or not infected. After 4 h of infection, HeLa cells were washed to remove bacteria and further cultivated. At 48 h postinfection, cells were washed and the cell morphology was analyzed by phase-contrast microscopy. Scale bars: 200 μm. (B) G_2_ cell cycle arrest. An increased number of sub-G_1_ cell populations (cell death) present after DNA damage were assayed by flow cytometry. (C) At 4 h postinfection, bacteria were removed and the cells were cultivated for another 4 h and subsequently washed with phosphate-buffered saline (PBS) and lysed. A total of 4 μg protein per lane of the indicated samples was analyzed by SDS-PAGE and afterwards transferred onto a polyvinylidene difluoride (PVDF) membrane. γ-H2AX was detected using anti-phospho-histone H2AX (Ser139) antibody (Millipore). β-Actin served as a loading control.

The *clbR* gene was originally annotated as a “putative transcriptional regulator” based on the significant similarity of the deduced ClbR amino acid sequence to sequences of transcription regulators of the LuxR/FixJ family (1, 33). The ClbR protein exhibits a high level of similarity to the transcription regulator GerE of Bacillus subtilis. Both GerE and ClbR contain a C-terminal helix-turn-helix (HTH) DNA-binding motif but lack an N-terminal regulatory receiver (REC) domain (34) (Fig. S2). Accordingly, and in contrast to many other LuxR/FixJ family members, both proteins are autonomous effector domain regulators and not response regulators. To gain the first insights into the role of ClbR in regulation of colibactin gene expression, we deleted *clbR* in E. coli M1/5 and compared the results seen with respect to cytopathic effect (CPE) and DNA damage in infected HeLa cells. In contrast to wild-type strain M1/5, deletion mutant M1/5 Δ*clbR* neither caused cell cycle arrest as shown by microscopic analysis and flow cytometry (Fig. 2A and B) nor increased levels of phosphorylated histone H2AX in HeLa cells (Fig. 2C). Complementation of E. coli M1/5 Δ*clbR* with pBAD-*clbR* restored the ability to block the cell cycle in HeLa cells as well as to induce the DNA damage cascade (Fig. 2).

10.1128/mSphere.00591-20.3 FIG S2 Comparison of the ClbR and GerE amino acid sequences. (A) Comparison of the ClbR and GerE amino acid sequence by Clustal Omega (88). (B) ClbR secondary structure prediction by JPred4 (89). (C) The ClbR monomer structure was modelled based on template 4HE1B. The GerE monomer structure has been resolved (1FSE2A). Download FIG S2, PDF file, 0.2 MB. Copyright © 2020 Wallenstein et al. 2020 Wallenstein et al. This content is distributed under the terms of the Creative Commons Attribution 4.0 International license.

Furthermore, we transformed previously described reporter strains of E. coli Nissle 1917 (EcN) carrying a transcriptional fusion of the *clbR* promoter, *clbA* promoter, *clbB* promoter, or *clbQ* promoter and the promoterless luciferase (*lux*) operon (33) with a plasmid which allows *clbR* expression under the control of the tetracycline-inducible promoter *tet*^p/o^. These strains were cultivated in lysogeny broth (LB), and levels of *clbR* promoter, *clbA* promoter, or *clbB* promoter activity in response to increased ClbR levels were compared by luminescence measurements. This experiment demonstrated that increased ClbR levels resulted in markedly increased promoter activities of the *clbR* and *clbB* genes, whereas the *clbA* promoter activity did not strongly respond to increased ClbR concentrations (Fig. S3). These results indicate that ClbR is a transcriptional (auto)activator of colibactin gene expression.

10.1128/mSphere.00591-20.4 FIG S3 ClbR induces transcription of *clbB* and *clbR*, but not of *clbA*, in E. coli strain Nissle 1917 (EcN). Plasmid-based *clbR* was overexpressed (37°C, LB, shaking conditions) in different EcN reporter strains carrying a chromosomal transcriptional *lux* fusion of different genes of the colibactin determinant that have been described before (33). The results indicate that *clbB* and *clbR* promoter activity increased significantly upon *clbR* overexpression, but not *clbA* promoter activity (unpaired two-tailed *t* test; *P* < 0.0005). (A) The data shown in the graph are mean values of results from biological triplicates. The error bars indicate standard deviations (SD). (B) Luciferase activity occurring in the different reporter strains in response to *clbR* overexpression (during growth on agar plates upon 16 h of incubation at 37°C) was also detected using a charge-coupled-device (CCD) camera. Download FIG S3, PDF file, 0.3 MB. Copyright © 2020 Wallenstein et al. 2020 Wallenstein et al. This content is distributed under the terms of the Creative Commons Attribution 4.0 International license.

### Promoter activity of *clbR* and colibactin expression depend on medium composition.

**FIG 3:**
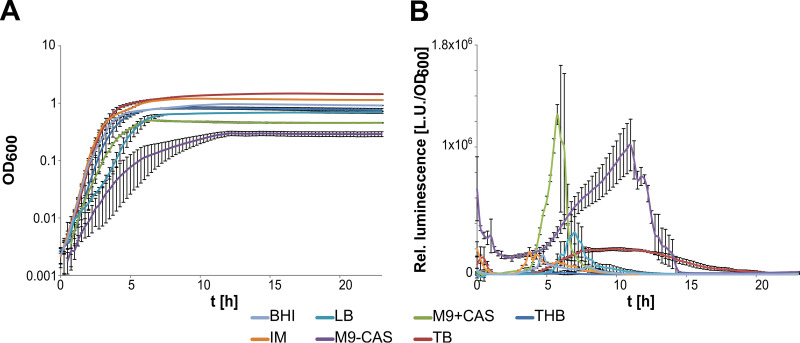
*clbR* promoter activity is dependent on growth phase and medium composition. The growth curves (A) and the corresponding relative luminescence levels (B) of the *clbR* promoter fusion in strain M1/5 *rpsL*K42R 5VNTR-p*clbR*-*lux* were compared during cultivation in different media (Todd Hewitt broth, THB; terrific broth, TB; M9 minimal medium with Casamino Acids, M9+CAS; M9 minimal medium without Casamino Acids, M9-CAS; lysogeny broth, LB; interaction medium, IM; brain heart infusion broth, BHI). Measurements were performed in biological and technical triplicates. The median luminescence values and standard deviations are shown.

To search for factors and conditions that affect *clbR* expression, we employed a reporter gene fusion based on the *clbR* promoter and the promoterless *lux* operon in E. coli M1/5. We tested different media such as lysogeny broth (LB), terrific broth (TB), M9 medium with and without Casamino Acids, interaction medium (IM), brain heart infusion (BHI), and Todd Hewitt broth (THB) (Fig. 3). Even though the use of each growth medium led to a characteristic pattern of *clbR* promoter activity, luminescence peaked during the transition from exponential growth to stationary phase except for IM and BHI media, where *clbR* promoter activity peaked during mid-exponential growth. Expression levels of *clbR* were higher in poorer than in richer media. The highest values for relative light units (RLU)/optical density at 600 nm (OD_600_) were observed upon bacterial cultivation in M9 media, and growth in M9 medium with Casamino Acids (M9+CAS) resulted in the most highly defined peak of *clbR* promoter activity. Cultivation in TB, THB, LB, and BHI medium resulted in a much lower expression level than growth in defined media, such as interaction medium and M9 minimal medium. We also constructed a reporter module based on the *frr* promoter as a “housekeeping reference” in E. coli M1/5. We measured the *clbR* and *frr* promoter activities in E. coli M1/5 upon growth in LB and in M9+CAS medium. The corresponding data are presented in Fig. S4. Under both growth conditions tested, the curve shapes were similar for *clbR* and *frr* promoter activities. However, only the *clbR* promoter activity and not the *frr* promoter activity increased significantly with growth in M9+CAS medium compared to LB. This supports our observation that *clbR* promoter activity is specifically induced in poorer media than in LB. On the basis of these findings, we decided to perform all further analyses of colibactin or ClbR expression in E. coli M1/5 in bacterial samples harvested in the late exponential growth phase in M9 medium with Casamino Acids.

10.1128/mSphere.00591-20.5 FIG S4 Comparison of levels of *clbR* promoter activity of E. coli strain M1/5 in LB and M9+CAS medium. Relative levels of luminescence (solid line, primary *y* axis) and growth (dashed line, secondary *y* axis) of E. coli M1/5 λ-*attB*::*cat*-*frr*p-*lux* (black) and M1/5 5VNTR-*clbR*p-*lux* (red) in LB medium (A) or in M9+CAS medium (B) are indicated. Median values of results from biological and technical triplicates are shown with SD. Download FIG S4, PDF file, 0.1 MB. Copyright © 2020 Wallenstein et al. 2020 Wallenstein et al. This content is distributed under the terms of the Creative Commons Attribution 4.0 International license.

To see whether the *clbR* promoter activity would respond to the growth medium and growth phase to the same extent as that seen with other E. coli isolates, we integrated the same the *clbR*p-*lux* reporter module used in E. coli M1/5 into the chromosomal λ-*attB* site of different model strains, in which colibactin expression has been studied previously, including probiotic strain Nissle 1917, uropathogenic strain UTI89, and newborn meningitis isolates IHE3034 and SP15. We then compared the levels of *clbR* promoter activity upon cultivation in M9+CAS medium or in LB in these strain backgrounds. In principle, the expression profiles seen with the *clbR* promoter were very similar to those seen in the other strain backgrounds such as E. coli M1/5; i.e., the promoter activity reached its maximum in the (late) logarithmic-growth phase (Fig. S5). In general, the promoter activity in LB was also significantly lower than in M9+CAS. Interestingly, there were also differences in the *clbR* promoter activity among the strains; the promoter activity was always higher in E. coli isolates Nissle 1917 and IHE3034 than in E. coli strains UTI89 and SP15. In particular, the levels of promoter activity seen with probiotic strain Nissle 1917 and fecal isolate M1/5 in M9+CAS were very similar (Fig. S5). The results obtained in different strain backgrounds showed that colibactin expression in E. coli reached its maximum in the late exponential-growth phase and was generally higher in poor media than in rich media. Despite basically uniform expression profiles, the levels of strength of *clbR* promoter activity may differ in different strain backgrounds.

10.1128/mSphere.00591-20.6 FIG S5 Comparison of levels of *clbR* promoter activity of different E. coli isolates. Relative levels of luminescence (solid lines, primary *y* axis) and growth (dashed lines, secondary *y* axis) of different E. coli strains carrying the λ-*attB*::5VNTR-*clbR*p-*lux* reporter module upon growth in LB (A) and M9+CAS medium (B) are indicated. Median values of results from biological and technical triplicates are shown with SD. Download FIG S5, PDF file, 0.7 MB. Copyright © 2020 Wallenstein et al. 2020 Wallenstein et al. This content is distributed under the terms of the Creative Commons Attribution 4.0 International license.

### Expression of *clbR* responds to iron availability.

**FIG 4:**
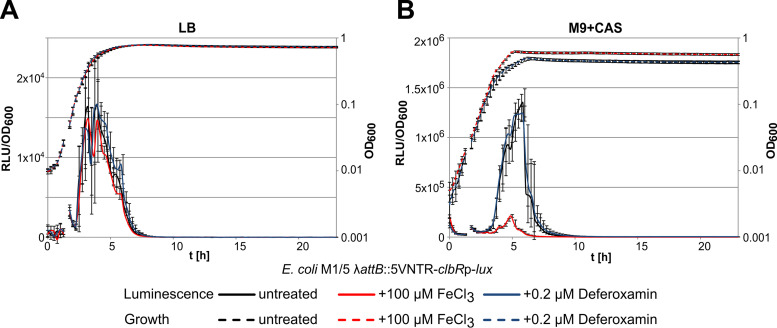
*clbR* promoter activity depends on iron availability. Levels of growth (OD_600_) and relative luminescence (RLU/OD_600_) of E. coli strain M1/5 λ-*attB*::5VNTR-*clbR*p-*lux* were measured in LB (A) and M9+CAS medium (B) depending on iron availability. The availability of ferric iron was altered by the addition of either 100 μM FeCl_3_ or 0.2 μM deferroxamin. Median values of results from biological and technical triplicates are shown with standard deviations.

Previous studies have shown that regulation of colibactin expression responds to iron availability via Fur-dependent and RyhB-dependent regulation of *clbA* transcription, thus affecting colibactin production (Tronnet et al. [35]). To find out whether expression of the main transcriptional activator of the colibactin genes is also regulated in response to iron availability, we employed the E. coli M1/5 λ-*attB*::5VNTR-*clbR*p-*lux* reporter strain described above to study *clbR* promoter activity under conditions of iron limitation or in the presence of an increased Fe(III) ion concentration. An increase in ferric iron availability in LB mediated by adding 100 μM FeCl_3_ as well as iron limitation mediated by adding 0.2 μM deferoxamine had no significant effect either on growth of the E. coli M1/5 reporter strain or on *clbR* promoter activity (Fig. 4A). Interestingly, addition of 100 μM FeCl_3_ to M9+CAS resulted in a strong reduction of *clbR* promoter activity, whereas the presence of 0.2 μM deferoxamine did not affect reporter gene expression during growth (Fig. 4B). These results indicate that ClbR expression is directly altered by the availability of iron also.

### ClbR interacts with the *clbR*-to-*clbB* intergenic region of the colibactin island.

**FIG 5:**
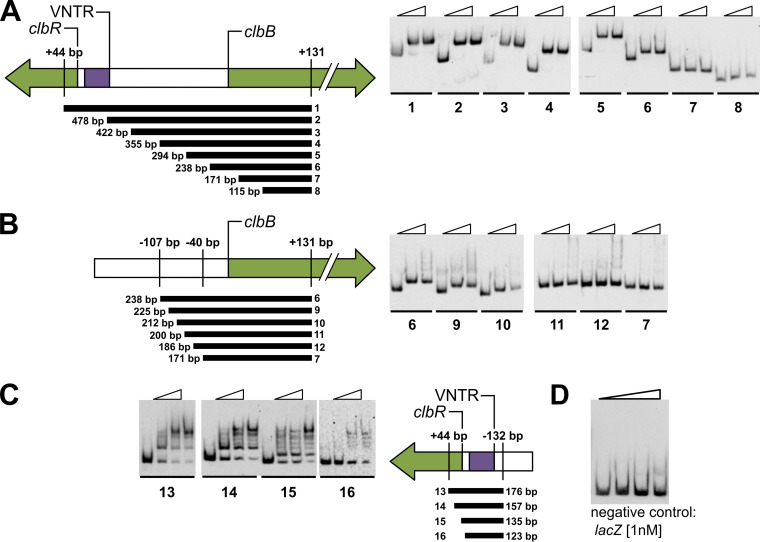
ClbR binds to *clbR* and *clbB* upstream regions. To demonstrate ClbR-DNA interactions using EMSA, PCR-generated, digoxigenin-labeled DNA fragments (300 pM) obtained from the upstream region of *clbR* and *clbB*, respectively, were incubated with increasing amounts of purified ClbR protein (for probes 1 to 12, 0 nM, 50 nM, or 100 nM ClbR per lane; for probes 13 to 16, 0 nM, 50 nM, 100 nM, or 150 nM ClbR per lane). The size and position of each of the probes are given relative to the translational start of *clbR* and *clbB*, respectively. (A to C) Probes 1 to 12 were used to narrow down the ClbR binding site upstream of *clbB* (A and B), and probes 13 to 16 were used to analyze ClbR binding to the *clbR* upstream region (C). Panels A and B refer to different subsets of probes tested for the *clbB* promoter region. (D) To confirm specific binding of ClbR, a negative control, i.e., a promoter fragment that lacks the ClbR binding motif, was included. For this purpose, a *lacZ* promoter-based probe [1 nM] was incubated with increasing amounts of purified ClbR protein (0 nM, 50 nM, 100 nM, and 200 nM ClbR per lane). The use of ClbR concentrations at which clear shifts were observed with probes representing the *clbR* or *clbB* promoter regions did not lead to reduced migration behavior of the *lacZ* probe.

To investigate whether ClbR interacts with DNA and to identify putative ClbR binding sites within the colibactin island, a series of electrophoretic mobility shift assays (EMSAs) were performed with purified ClbR, focusing on the intergenic region between *clbB* and *clbR*. With DNA probes of decreasing sizes, we scanned the *clbB-R* intergenic region for those parts which interact with the ClbR protein. We identified a ClbR binding site close to *clbB* and found that the binding motif is located between position bp −40 and position bp −107 upstream of the *clbB* translational start, since no interaction of ClbR with probes 7, 8, 11, and 12 was detected (Fig. 5A and B). We also studied the interaction of ClbR with the immediate upstream region of its own coding sequence by scanning a 123-bp region upstream of *clbR*, including the VNTR region, with DNA probes of differing size. ClbR interaction with probes generated from the *clbR* upstream region were observed with probes 13, 14, and 15 but were no longer observed with probe 16 (Fig. 5C). Accordingly, the DNA stretch upstream of position −2 relative to the *clbR* translational start is required for ClbR binding. In contrast to the clean shift observed in EMSAs performed with the *clbB* upstream fragment, the region close to *clbR* exhibited more-complex interactions with ClbR (Fig. 5C). The *clbR* gene is preceded by a VNTR region (Fig. 1), and such regions can differ in size. Between 2 and 20 repeats have been described so far (2). We assume that the VNTR region or the overall tertiary structure of this DNA stretch interfered with efforts to reveal a clearer assessment of the migration behavior of the probes designed for this part of the intergenic region between *clbB* and *clbR*. Purified ClbR protein did not interact with the probe representing the *lacZ* promoter region that served as a negative control (Fig. 5D). Accordingly, we have demonstrated that ClbR can interact with the *clbB-R* intergenic region. We narrowed down the DNA stretch in *clbB* and also that in the *clbR* upstream region to which ClbR binds. Our results corroborate the predicted function of ClbR as transcriptional regulator and (auto)activator.

### ClbR binding regions and overlapping of *clbR* and *clbB* promoter regions.

**FIG 6:**
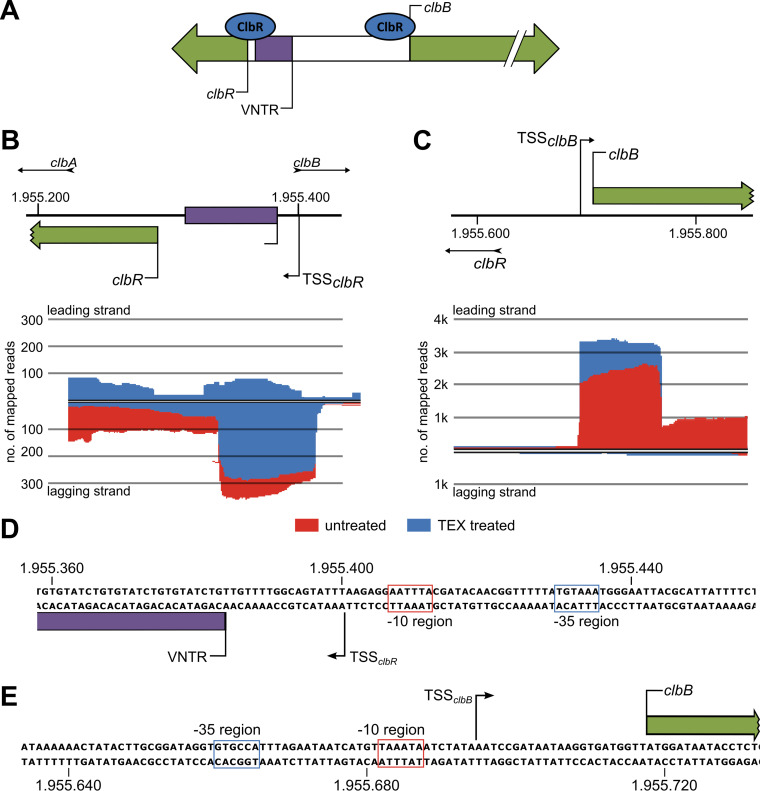
The ClbR binding regions overlap the transcriptional start sites of *clbR* and *clbB*. (A) ClbR binds to the upstream regions of *clbR* and *clbB*. (B) To further characterize the role of ClbR as a transcriptional activator of *clbR* and *clbB*, we determined the transcriptional start sites of both genes in E. coli M1/5 *rpsL*K42R by differential RNA-seq. By comparing mapped sequencing reads of TEX-treated (blue) and untreated (red) RNA samples, the transcriptional start site (TSS) of *clbR* was identified upstream of the VNTR region. (C) Using the same method as that described for panel B, we determined the *clbB* transcriptional start upstream of the *clbB* translational start site. (D and E) The corresponding nucleotide sequence and predicted promoter elements of the transcriptional start sites of *clbR* (D) and *clbB* (E) are indicated.

To further characterize the putative role of the VNTR region as a regulatory element located in the *clbR-B* intergenic region (Fig. 6A), we investigated the exact transcription start site (TSS) of *clbR* and *clbB* by differential transcriptome sequencing (RNA-seq) and compared mapped sequencing reads of untreated and terminator 5′-phosphate-dependent terminator exonuclease (TEX)-treated (enriched for primary transcripts) RNA samples isolated from E. coli strain M1/5 (Fig. 6B and C). Judging on the basis of the number of sequence reads mapped to the chromosomal region close to *clbR*, this gene is only weakly transcribed. We identified the *clbR* TSS start 16 bp upstream of the VNTR region, suggesting that this stretch of repeats belongs to the 5′ untranslated region of *clbR* (Fig. 6D). In contrast, *clbB* is much more strongly expressed at the transcriptional level than *clbR* (Fig. 6B and C), and the *clbB* transcriptional start site was mapped to position −24 relative to the *clbB* translational start (Fig. 6D). The EMSA and differential RNA-seq data demonstrate that the *clbR* and *clbB* transcription start sites overlap the ClbR binding regions within the intergenic region between these two genes (Fig. 5; see also Fig. 6A).

### The VNTR region affects *clbR* expression at the transcriptional level.

**FIG 7:**
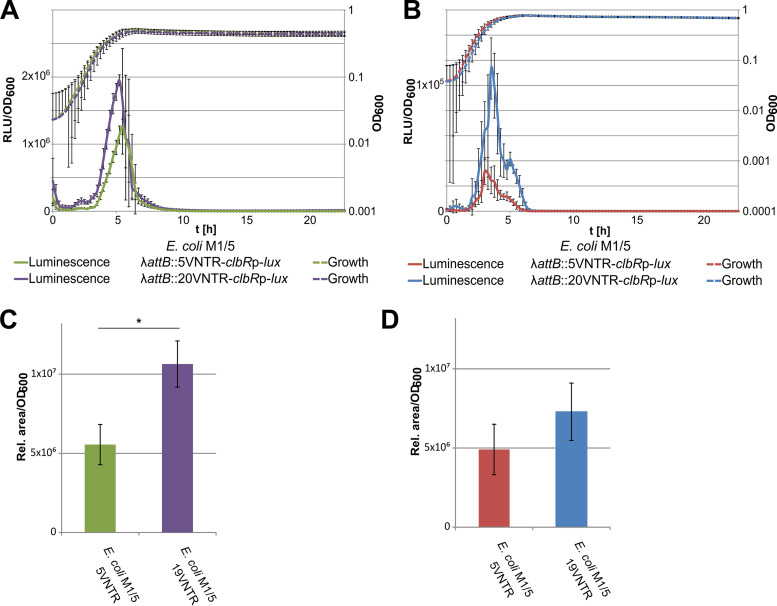
The size of the VNTR region affects colibactin production via altered *clbR* transcription. The VNTR region is part of the untranslated 5′ region of the *clbR* transcript. (A and B) As VNTR regions of various sizes have been observed in different E. coli isolates, we tested the impact of five VNTRs versus 20 VNTRs on *clbR* promoter activity by the use of λ-*attB* site-inserted luciferase reporter fusions in E. coli strain M1/5 *rpsL*K42R grown in M9 medium supplemented with Casamino Acids (A) and in LB (B). Measurements were performed in biological and technical triplicates, and representative graphs are shown. (C and D) We also measured the impact of the size of the VNTR region and of M9 medium (C) or LB (D) on colibactin production of E. coli M1/5 *rpsL*K42R with altered numbers of VNTRs in the native VNTR site via quantification of the precolibactin cleavage product N-myristoyl-d-asparagine (C14-Asn). The data presented in the graphs were obtained from three biological replicates. *, *P* < 0.05, unpaired *t* test.

To analyze whether a VNTR region of a different size would affect *clbR* expression, we inserted luciferase-based reporter constructs, which are fused to the *clbR* upstream region with a VNTR region of either 5 or 20 repeats, into the chromosomal attachment site of bacteriophage λ (λ-*attB*) and tested for luciferase expression. We measured 2-fold-higher luminescence with the 20-repeat VNTR region than with the VNTR region containing 5 repeats (Fig. 7A and B), suggesting a regulatory impact associated with this particular DNA stretch. We conclude from our reporter gene studies that *clbR* transcript levels depend on growth phase, resource availability, and length of the VNTR region.

As colibactin expression correlates with the level of available ClbR, we also assessed colibactin production changes in response to the growth medium or size of the VNTR region by quantifying C14-Asn as a by-product of colibactin biosynthesis. For this purpose, the repeat number of the VNTR region had to be modified by scarless mutagenesis, and we generated variants of the native VNTR region comprising either 5 or 19 repeats. The C14-Asn levels produced by isogenic E. coli M1/5 variants carrying a VNTR region with either 5 or 19 repeats upstream of *clbR* supported our observations made with the chromosomal λ-*attB* site-inserted luciferase-based reporter fusions comprising 5 or 20 VNTRs. The concentration of C14-Asn increased with increasing VNTR region size and was also higher upon cultivation in M9 medium supplemented with Casamino Acids than in LB (Fig. 7C and D). As a result, our data show that *clbR* expression is modulated by the composition of the growth medium and by the size of the VNTR region located in the 5′ untranslated region of *clbR*.

### Modulation of colibactin expression via transcriptional activation or altered performance of the production machinery.

**FIG 8:**
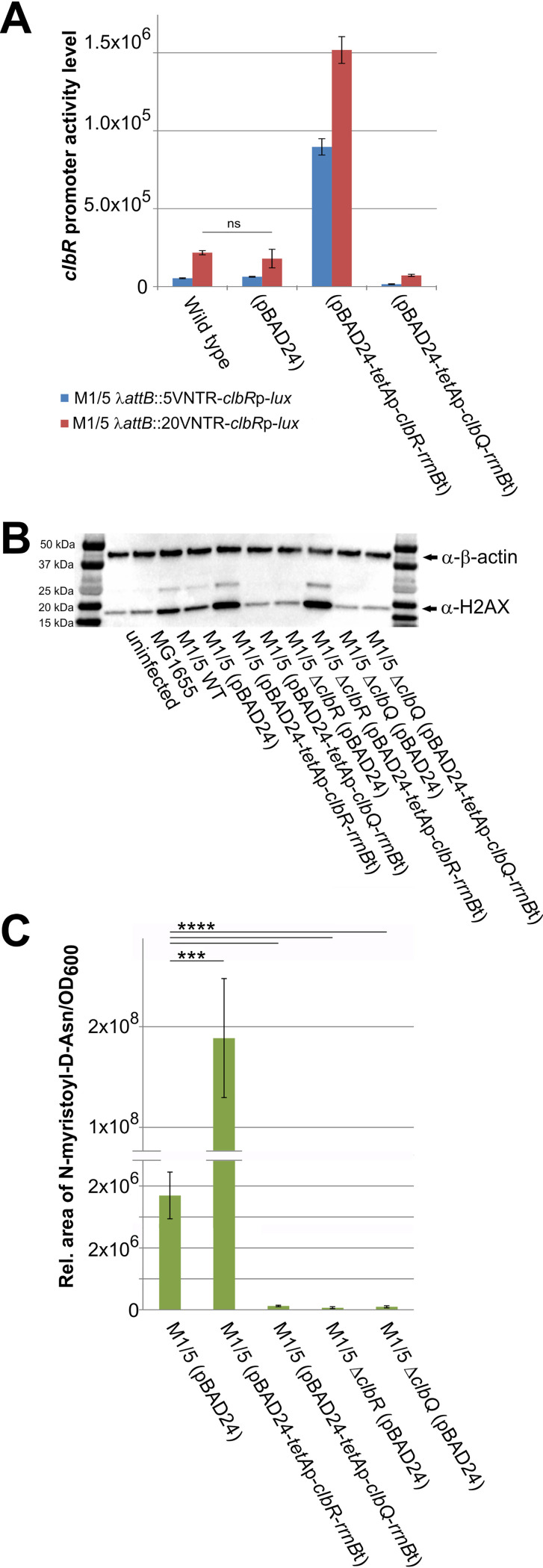
ClbR and ClbQ levels alter colibactin-mediated phenotype in cell culture assays. The impact of ClbR and ClbQ on *clbR* expression and colibactin production was tested. (A) E. coli strain M1/5 *rpsL*K42R carrying a chromosomally λ-*attB* site-inserted *clbR* promoter-luciferase fusion that included either a 5-repeat or 20-repeat VNTR region was transformed with pBAD24 derivatives, enabling overexpression of *clbR* or *clbQ*. Luminescence as a measure of *clbR* promoter activity was quantified in response to increased expression of *clbR* and *clbQ*. Data are based on results from three biological replicates performed with three technical replicates. Means with standard deviations are shown. Except for E. coli M1/5 *rpsL*K42R with and without the vector control, the *clbR* promoter activities measured differed significantly in response to *clbR* and *clbQ* overexpression (*P* > 0.0001, unpaired *t* test). (B) HeLa cells were either infected with E. coli strain M1/5 *rpsL*K42R or derivatives (MOI of 200) or not infected. At 4 h postinfection, bacteria were removed and the cells were cultivated for another 4 h and subsequently washed with PBS and lysed. A total of 6 μg protein per lane of the indicated samples was analyzed by SDS-PAGE and afterwards transferred onto a PVDF membrane. γ-H2AX was detected using anti-gammaH2A.X (phospho S139) antibody (Abcam). β-Actin served as a loading control. Corresponding bands are marked with an arrow. For colibactin-producing strains, the ubiquitinylated band (∼25 kDa) could also be detected. (C) The impact of ClbR and ClbQ on colibactin production of M1/5 *rpsL*K42R was also analyzed by UPLC-HRMS-based comparison of N-myristoyl-d-asparagine levels. The data presented in the graph were obtained from three biological replicates. Mean values with standard deviations are shown. ****, *P* < 0.0001; ***, *P* < 0.001 (unpaired *t* test).

To further investigate the role of ClbR as a key regulator of colibactin production as well as of general regulatory aspects of the *pks* island, we decided to compare the levels of colibactin production seen upon deletion or overexpression of *clbR*, i.e., in the absence of transcriptional activation or full induction of transcription of the colibactin determinant. Additionally, we tested whether colibactin production is subject to feedback regulation and therefore analyzed the levels of colibactin production seen upon deletion or overexpression of *clbQ*. ClbQ encodes a type II-family editing thioesterase, which controls the flux of substrates and intermediates during colibactin biosynthesis as well as the overall performance of the production machinery (26). Luminescence measurements performed with different chromosomally inserted reporter constructs that enable analysis of *clbR* promoter activity supported our finding that overexpression of *clbR* in *trans* resulted in increased *clbR* promoter activity whereas *clbQ* overexpression in *trans* had no drastic effect on *clbR* promoter activity. Furthermore, these luciferase assays also convincingly demonstrate that the size of the VNTR region upstream of *clbR* promoter affected the *clbR* transcription level (Fig. 8A). Infection of HeLa cells followed by indirect assessment of colibactin expression via quantification of phosphorylated histone γ-H2AX indicated that *clbR* overexpression in E. coli strain M1/5 led to a strong increase of γ-H2AX levels, whereas γ-H2AX levels were markedly decreased in the *clbR* deletion mutant relative to the wild-type strain (Fig. 8B). In contrast, overexpression of *clbQ* as well as *clbQ* deletion in E. coli M1/5 reduced the detectable amount of γ-H2AX in infected HeLa cells. Complementation of E. coli M1/5 Δ*clbR* with pBAD24-*tetA*p-*clbR*-*rrnB*t and complementation of the *clbQ* deletion mutant of E. coli M1/5 with pBAD24-*tetA*p-*clbQ*-*rrnB*t resulted in γ-H2AX levels in infected HeLa cells that corresponded to those observed upon infection with M1/5 derivatives overexpressing *clbR* and *clbQ*, respectively (Fig. 8B). UPLC-HRMS (ultra-high-performance liquid chromatography–high resolution mass spectrometry) measurements of the colibactin biosynthetic by-product C14-Asn confirmed that colibactin production was significantly increased upon overexpression of *clbR*, whereas *clbR* deletion, but also *clbQ* overexpression and *clbQ* deletion, abolished colibactin expression (Fig. 8C). These results do not support the idea of a potential form of feedback regulation of colibactin gene expression but rather suggest that modulation of the ClbQ protein level reduced the overall performance of the colibactin production machinery or the level of intermediates of the colibactin biosynthesis process.

### General impact of colibactin production on the E. coli M1/5 transcriptome and proteome.

To analyze expression of the colibactin determinant at the transcriptomic or proteomic level, and to find out to what extent expression of the colibactin island is integrated into regulatory and metabolic networks, we compared the transcriptome and proteome of E. coli strain M1/5 with those of M1/5 mutants lacking or overexpressing *clbR* as well as those of the *clbQ* deletion and overexpressing mutants. In this way, we also aimed to identify ClbR-dependent determinants located outside the *pks* island as well as candidate genes whose expression might be affected by the activity of the colibactin production machinery or by the availability of metabolites and intermediates related to colibactin production.

**FIG 9:**
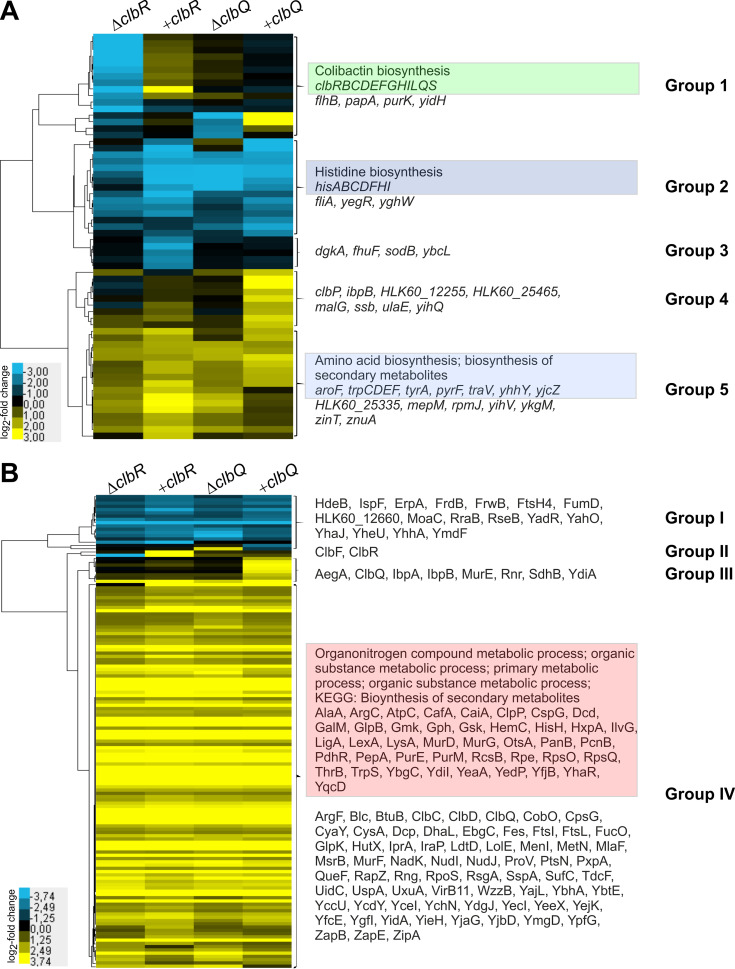
Impact of *clbR* and *clbQ* expression on global gene expression of E. coli M1/5 *rpsL*K42R at the transcriptome and proteome levels. We compared the levels of impact of *clbR* or *clbQ* overexpression as well as of *clbR* or *clbQ* deletion on global gene expression of E. coli M1/5 *rpsL*K42R at the transcriptional and translational levels by RNA-seq and proteome analysis, respectively. Three biological replicates were pooled before aliquots were used for RNA-seq or mass spectrometry-based proteome analysis. The expression profiles of genes (A) and proteins (B) displaying a log_2_ fold change value of less than or equal to −2 or greater than or equal to +2 in the different E. coli M1/5 variants relative to the wild type were subjected to cluster analysis. The gene/protein designations are indicated as well as groups of genes/proteins with similar expression patterns. Groups of genes or proteins that displayed an enrichment of functionally related proteins are marked in color, and the corresponding Gene Ontology (GO) term is given.

To screen for candidate genes which are markedly deregulated in E. coli M1/5 in response to different levels of available ClbR or ClbQ proteins, we pooled three biological replicates of E. coli strain M1/5 or its corresponding mutants and either isolated total RNA for differential RNA-seq analysis or performed gel-free proteomics to analyze the protein content of whole bacterial cells. Transcript levels of only 62 genes were deregulated in at least one of the *clbR* or *clbQ* mutants relative to wild-type strain M1/5 with a log_2_ fold change value less than or equal to −2 or greater than or equal to +2 (Fig. 9A; see also Table S2). Clustering of deregulated genes identified five groups of genes with different expression profiles in the four strains (Fig. 9A). Whereas transcription of the individual genes of the colibactin gene cluster (group 1) was downregulated in the *clbR* deletion mutant, it was upregulated upon overexpression of *clbR* (Fig. 9A; see also Fig. S6A and B). The transcriptomic data for individual genes of the colibactin determinant are in good agreement with quantitative reverse transcription-PCR (qRT-PCR) results for *clbA*, *clbR*, *clbB*, and *clbQ* (Fig. S6A). Apart from that, the transcript levels of group 2 genes involved in histidine biosynthesis were more strongly repressed upon overexpression of *clbR* and deregulation of *clbQ* expression than in the *clbR* mutant. In contrast, transcription of the group 3 genes, which comprise the two iron-regulated *sodB* and *fhuF* genes, was specifically reduced in response to *clbR* overexpression. Deletion and overexpression of *clbQ* had only a weak effect on transcript levels of the genes in the colibactin gene cluster, except for *clbQ* and *clbS* (Fig. S6A and D) but markedly affected, among others, the transcript levels of genes involved in amino acid and secondary metabolite biosynthesis (Fig. 8A, gene groups 2, 4, and 5). Although the transcriptome profiles of strains overexpressing *clbR* or *clbQ* could be distinguished from those of the *clbR* or *clbQ* deletion mutants, modulation of availability of both ClbR and ClbQ in E. coli M1/5 had similar overall impacts on gene expression at the transcriptional level. Whereas expression of genes involved in histidine biosynthesis was markedly repressed under all four tested conditions, genes required for biosynthesis of secondary metabolites and for aromatic amino acid (tryptophan, tyrosine, phenylalanine) biosynthesis or metabolization were upregulated (Fig. 8A).

10.1128/mSphere.00591-20.7 FIG S6 Impact of *clbR* and *clbQ* deletion or *clbR* and *clbQ* overexpression on the expression level of the colibactin determinant in E. coli M1/5. Transcript levels were compared by qRT-PCR. (A) Median values of results from biological triplicates are shown with corresponding standard deviations. Transcript levels were compared by RNA-seq and protein levels were compared by mass spectrometry. (B to E) Panels B and D compare fold changes in transcript levels based on DESeq2 analysis, whereas panels C and E compare fold changes in normalized protein abundances depending on ClbR (B and C) or ClbQ (D and E) availability. Download FIG S6, PDF file, 0.2 MB. Copyright © 2020 Wallenstein et al. 2020 Wallenstein et al. This content is distributed under the terms of the Creative Commons Attribution 4.0 International license.

10.1128/mSphere.00591-20.9 TABLE S2 Differentially expressed genes and proteins in E. coli M1/5 in response to altered *clbR* or *clbQ* expression. Download Table S2, PDF file, 0.3 MB. Copyright © 2020 Wallenstein et al. 2020 Wallenstein et al. This content is distributed under the terms of the Creative Commons Attribution 4.0 International license.

In addition, we compared the proteomes of these mutants and identified 145 proteins with log_2_ fold change values of less than or equal to −2 or greater than or equal to +2 (Fig. 8B; see also Table S2). For six identified proteins, we were also able to describe the corresponding genes as deregulated by RNA-seq. In five cases (ClbR, ClbC, ClbD, ClbF, and ClbQ), these were proteins/genes involved in colibactin biosynthesis. Only one of six deregulated genes (*ibpB*) and its encoded protein, IbpB, were not directly linked to the colibactin production machinery. As expected, ClbR and ClbQ protein levels were markedly increased upon overexpression of *clbR* and *clbQ*, respectively. While the expression levels of most detected Clb proteins did not strongly respond to the availability of ClbQ, some Clb proteins exhibited opposite levels of expression according to whether *clbR* was deleted or overexpressed (Fig. S6C and E). The global protein expression profiles determined for the deletion of *clbR* and *clbQ* as well as for the mutants overexpressing *clbR* and *clbQ* were very similar at the protein level also. Cluster analysis indicated that a small number of (group I) proteins was repressed in the four mutants relative to the wild type. The majority of detected deregulated (groups III and IV) proteins were upregulated in the four mutants compared to the wild type (Fig. 8B). Gene Ontology (GO) analysis using E. coli strain CFT073 as reference allowed classification of 38 of 62 deregulated genes and 112 of 145 deregulated proteins detected in the E. coli M1/5 background into functional networks (Table 1). It is interesting that modulation of colibactin production by deletion and by overexpression of either *clbR* or *clbQ* had similar effects on gene expression (at the transcriptional or protein level). Although the results of the transcriptome and proteome analyses overlap in only six genes/gene products, the results show that secondary metabolite biosynthesis is influenced by the activity of the colibactin biosynthesis machinery. Several related groups of genes or gene products involved in amino acid (histidine, phenylalanine, tyrosine, and tryptophan) biosynthesis or organonitrogen compound and primary metabolic processes, which are in principle also relevant for polyketide biosynthesis, were deregulated at either the transcriptome or the proteome level. In summary, our observation that the clearly contrasting forms of regulation seen upon *clbR* deletion and overexpression were detectable only for the colibactin gene cluster supports our view that ClbR is the specific key transcriptional regulator of the colibactin determinant. Our global gene expression data also indicate that colibactin production is functionally connected to pathways involved in biosynthesis or metabolization of particular amino acids and secondary metabolites.

**TABLE 1:** GO term and KEGG pathway analysis of differentially regulated genes and proteins in E. coli M1/5 *rpsL*K42R in response to different *clbR* or *clbQ* expression levels

Gene or protein	Description	Genes present (%)	False-discovery rate
Deregulated genes			
GO biological process			
GO:0000105	Histidine biosynthetic process	77.8	2.08E−08
GO:0006547	Histidine metabolic process	77.8	2.08E−08
GO:0052803	Imidazole-containing compound metabolic process	77.8	2.08E−08
GO:0008652	Cellular amino acid biosynthetic process	8.9	6.35E−07
GO:1901607	Alpha-amino acid biosynthetic process	9.2	1.71E−06
GO:0009073	Aromatic amino acid family biosynthetic process	25.0	1.98E−05
GO:0009072	Aromatic amino acid family metabolic process	21.4	3.74E−05
GO:1901605	Alpha-amino acid metabolic process	5.7	8.35E−05
GO:0006520	Cellular amino acid metabolic process	4.8	0.00013
GO:1901566	Organonitrogen compound biosynthetic process	3.2	0.00013
GO:0000162	Tryptophan biosynthetic process	44.4	0.00017
GO:0006568	Tryptophan metabolic process	36.4	0.00026
GO:0044283	Small-molecule biosynthetic process	3.8	0.00035
GO:0019438	Aromatic compound biosynthetic process	2.7	0.00087
GO:1901362	Organic cyclic compound biosynthetic process	2.5	0.0017
GO:0006082	Organic acid metabolic process	2.8	0.0018
GO:0044281	Small-molecule metabolic process	2.2	0.004
GO:0044249	Cellular biosynthetic process	1.9	0.0044
GO:1901576	Organic substance biosynthetic process	1.9	0.0049
GO:0034224	Cellular response to zinc ion starvation	100.0	0.0082
GO:0019752	Carboxylic acid metabolic process	2.6	0.01
GO:0018130	Heterocycle biosynthetic process	2.3	0.0119
GO:1901564	Organonitrogen compound metabolic process	2.0	0.0119
GO:0061720	6-Sulfoquinovose(1-) catabolic process to glycerine phosphate and 3-sulfolactaldehyde	50.0	0.0168
GO:1902776	6-Sulfoquinovose(1-) metabolic process	33.3	0.0279
GO:0044238	Primary metabolic process	1.4	0.0436
GO:0006725	Cellular aromatic compound metabolic process	1.7	0.0497
KEGG pathway			
eco00340	Histidine metabolism	87.5	6.85E−10
eco01230	Biosynthesis of amino acids	10.3	1.47E−08
eco00400	Phenylalanine, tyrosine, and tryptophan biosynthesis	28.6	1.17E−06
eco01110	Biosynthesis of secondary metabolites	4.7	3.71E−06
eco01100	Metabolic pathways	2.4	0.00087
eco00401	Novobiocin biosynthesis	50.0	0.0057
eco01130	Biosynthesis of antibiotics	3.3	0.0146

Deregulated proteins			
GO biological process			
GO:1901564	Organonitrogen compound metabolic process	6.1	1.49 × 10^−5^
GO:0071704	Organic substance metabolic process	4.8	1.49 × 10^−5^
GO:0044238	Primary metabolic process	4.8	1.49 × 10^−5^
KEGG pathway			
eco00523	Polyketide sugar unit biosynthesis	50.0	0.0325
eco00550	Peptidoglycan biosynthesis	21.7	0.0325
eco01100	Metabolic pathways	4.8	0.0325
eco01110	Biosynthesis of secondary metabolites	6.3	0.0325
eco01130	Biosynthesis of antibiotics	7.7	0.0325
eco00521	Streptomycin biosynthesis	33.3	0.0404

## DISCUSSION

Although our knowledge of the colibactin biosynthetic mechanism and of the molecular structure of this nonribosomal peptide/polyketide and its mode of action is continuously increasing (11, 19, 20, 22–24, 26, 27, 36, 37), we still have little information on the mechanisms of its regulation. So far, colibactin production and regulation of colibactin gene expression have been mainly analyzed in the context of DNA damage and genotoxicity (1, 8, 9), extraintestinal pathogenic E. coli (ExPEC) pathogenesis (6, 7) but also as factors contributing to the probiotic character of E. coli strain Nissle 1917 (3, 4). Model strains used for the functional characterization of factors involved in colibactin expression are human clinical isolates of ST95 (E. coli O18:K1 newborn meningitis strains IHE3034 and SP15) (1, 35), ST73 (uropathogenic E. coli isolate CFT073) (22), probiotic E. coli strain Nissle 1917 (ST73) (33, 36), or laboratory strain E. coli DH10B (pBeloBAC11-*pks*) (24, 38, 39). Many E. coli human or murine isolates used for *in vivo* models of colibactin function have not been characterized in detail at the genomic level (12, 13, 40). To extend the spectrum of well-characterized model strains, we present here the complete genome sequence of human fecal E. coli isolate M1/5, which was isolated from a healthy human individual. This strain represents the large group of colibactin-positive intestinal colonizers of phylogroup B2 without the increased pathogenic potential of ExPEC. E. coli M1/5 (O75:K5:H5) belongs to ST550/CC14 and does not express (cyto)toxins, which can interfere with the phenotypic analysis of colibactin expression in cell culture experiments. This strain also lacks other important E. coli virulence factors, such as type 3 secretion systems as well as typical virulence-related fimbrial adhesins of intestinal and extraintestinal E. coli pathotypes. Comparison of the genome content of E. coli M1/5 with that of other completely sequenced human commensal model strains frequently used for comparative genomics or functional analyses, i.e., E. coli isolates HS (phylogroup A, O9:H4; BioProject accession no. PRJNA13959) (41), SE11 (phylogroup B1, O152:H28; PRJNA18057) (42), IAI1 (phylogroup B1, O8:H19; PRJNA33373) (43), SE15 (phylogroup B2, O150:H5; PRJDA19053) (44), and ED1a (phylogroup B2, O81:H27; PRJNA33409) (43), indicated that these strains differ with respect to the presence of determinants for chaperone-usher fimbriae and other adhesins and of factors involved in serum resistance and iron uptake as well as in gene clusters coding for type 3, type 5, and type 6 secretion systems. E. coli M1/5 is the only isolate among these commensals that carries the colibactin as well as two different flagellar determinants (Flag-1 and Flag-2). Compared to the aforementioned fecal isolates from healthy individuals, the E. coli M1/5 genome comprises the highest number of detected genes that may contribute to fitness of extraintestinal pathogenic E. coli (see Table S1 in the supplemental material).

Our analyses indicate that ClbR is the main transcriptional activator specifically regulating colibactin biosynthesis. ClbR expression directly correlates with the production of functional colibactin. The *clbR* transcriptional profiles in five fecal and clinical E. coli model isolates were in principle very similar but also exhibited strain-specific differences. The molecular reasons for the different levels of *clbR* transcription, in particular, the reasons for the perception of the possible presence of inducing or repressing stimuli and their transmission through the interaction of different regulatory elements, are still not understood. It was reported previously that colibactin expression is affected by the bacterial growth state and the composition of the growth medium (33). We also know that the availability of spermidine and other polyamines is required for colibactin production via an as-yet-unknown regulatory mechanism (45). Our reporter gene-based analysis of *clbR* promoter activity suggests a form of resource-dependent and growth phase-dependent regulation, reflecting distinct *clbR* expression patterns with varying promoter activities. In most cases, the highest peaks of *clbR* promoter activity were detected at the transition from late exponential phase to early stationary phase and in less-complex media (Fig. 3; see also Fig. S3 in the supplemental material). Shifts of the colibactin gene expression peak were also observed in our previous study analyzing the impact of different carbon sources on transcription of the colibactin determinant in E. coli strain Nissle 1917 (33). This suggests that transcription of the colibactin determinant is regulated at least in part in response to the availability of metabolites and/or the activity of the central carbon metabolism. The fact that growth in TB, in contrast to other rich media, led to a significantly longer phase of *clbR* transcription which extended far into the stationary growth phase (Fig. 3) further illustrates that multiple parameters, such as the supply of nutrients and energy, are integrated into the regulation of colibactin expression. This integration is probably achieved by the action of regulators within the framework of regulatory networks. Screenings of random transposon insertion libraries of colibactin-producing strains have not yet identified any regulatory protein that could be involved in adjustment of colibactin expression in response to changing growth conditions.

Importantly, a direct impact on colibactin production via regulation of *clbA* gene expression has been reported for iron via the ferric uptake regulator (Fur) protein and the RyhB small regulatory RNA (35, 46). Here, we show that expression of the main transcriptional activator of the colibactin determinant is directly affected by iron availability also (Fig. 4). The exact mechanism responsible for iron-dependent regulation of *clbR* remains to be elucidated. While Fur binding sites have been detected upstream of *clbA* (35, 46), we did not identify such regions upstream of *clbR*. The fact that *clbR* promoter activity was unaffected by increased or decreased iron availability in LB, whereas addition of ferric chloride led to a drastic decrease of *clbR* promoter activity in minimal medium, may suggest that iron availability rather than availability of nutrients determines the level of *clbR* expression. Iron and nutrients are highly abundant in rich LB medium such that a decrease or increase in the iron concentration has no effect on *clbR* expression. In contrast, addition of ferric iron to the minimal medium poorer in iron (and nutrients) led to a strong reduction of *clbR* promoter activity. A further decrease of iron availability in minimal medium upon addition of the chelator deferoxamine had no effect. This finding suggests that, at least in the LB and M9+CAS medium investigated here, iron availability plays a more important role in the regulation of *clbR* transcription than nutrient supply. Our results are in accordance with other published data: Transcriptomic analyses indicate that colibactin expression is (at least at the transcriptional level) increased in E. coli upon growth in (iron-limited) urine relative to LB and is detectable during colonization of the intestinal tract and that intestinal inflammation promotes colibactin expression (47–49). Colibactin is considered a virulence factor of newborn meningitis-causing E. coli during sepsis (7) and seems to be important for long-term intestinal colonization (50). A form of regulation of colibactin expression that responds to iron availability in different body niches may support bacterial fitness in the blood or in the context of an Fe(III) ion gradient between the intestinal lumen and the intestinal epithelium, because it ensures fine-tuned colibactin expression under appropriate conditions. The close connection between the metallophore yersiniabactin and colibactin, on both the genomic and regulatory levels (51), is clearly underlined by the iron-dependent regulation of colibactin expression. Deeper insights into the structural diversity of molecules derived from the colibactin pathway, relevant growth conditions, and regulatory mechanisms will help us to better understand the biological role of this interesting and controversial secondary metabolite, whose production has been described to promote cancer but also to be associated with the probiotic character of E. coli (3, 4, 13, 16, 52–57).

ClbR contains a LuxR-type DNA-binding helix-turn-helix (HTH) domain in the C-terminal region which is usually found in response regulators of the LuxR/FixJ family. Classical LuxR/FixJ response regulators possess an N-terminal receiver (REC) domain. This REC domain is responsible for the activation of the response regulator (i) upon phosphorylation by a transmembrane sensor kinase (58), (ii) upon binding of N-acyl homoserine lactones (59, 60), or (iii) upon binding of multiple ligands (MalT) (61). A LuxR-like response regulator lacking a REC domain has been described previously also: transcription factor GerE regulates transcription of spore coat genes in the late sporulation stage in B. subtilis (34, 62). LuxR-type regulators are usually transcriptional activators, although some can act as repressors or, like GerE, can act as both activators and repressors (58). On the basis of its amino acid sequence and predicted structure, ClbR resembles the GerE protein (Fig. S2). As ClbR and GerE lack an N-terminal regulatory REC domain, it is unlikely that ligand binding results in activation of both regulatory proteins. Expression of GerE is regulated at the transcriptional level by a hierarchical cascade involving two different sigma factors and different levels of regulation, including transcription, DNA recombination, and proprotein processing (63). The molecular mechanism responsible for the growth phase-dependent and metabolite-dependent regulation of *clbR* expression may be as complex as that for GerE and remains to be characterized.

We demonstrated that ClbR binds to an intergenic region that separates the two divergently oriented gene clusters involved in regulation and activation of colibactin expression (*clbR* and *clbA*) or in biosynthesis and delivery of the polyketide (*clbB* to *clbS*) (Fig. 1, top panel). This intergenic region comprises the promoters of *clbR* and *clbB* as well as an additional regulatory element, i.e., the VNTR region, which is located upstream of the *clbR* translational start site (Fig. 1, bottom panel; see also Fig. 5A). Our results indicate that the size of the VNTR region affects *clbR* promoter activity and thus colibactin production (Fig. 7 and 8). We assume that the number of repeats and thus the size and secondary structure of the *clbR* 5′ region can affect *clbR* transcription as well as transcript stability and the efficacy of translation.

ClbR seems to be a *pks* island-specific regulator, because our transcriptome analysis in *clbR* deletion and overexpressing mutants did not indicate that transcription of other genes located outside the *pks* island was directly dependent on ClbR availability. Only transcription of the *clb* gene cluster (group 1 genes) changed accordingly with the deletion or overexpression of *clbR* (Fig. 8A; see also Fig. S6A and B). Otherwise, the few genes which exhibited deregulation in response to deletion or overexpression of *clbR* (Fig. 8A, group 2 to group 5) responded in fairly similar manners to *clbQ* deletion and overexpression also. Only the members of a small group of genes (comprising group 1 and group 4), including *clbP* and *clbQ*, were upregulated upon *clbQ* overexpression (Fig. 8A). For cases in which some genes/gene products were able to be clustered according to function, these functionally associated groups are highlighted in Fig. 8. The fact that deletion and overexpression of *clbR* and *clbQ* led to very similar global expression profiles at the transcriptome and proteome levels (Fig. 8), in particular, the expression profiles of those genes whose products can be functionally associated with amino acid (histidine, phenylalanine, tyrosines, and tryptophan) and secondary metabolite biosynthesis but also with organonitrogen compound and primary metabolism (gene groups 2 and 5, protein group IV), suggests that these processes are indirectly affected by ClbR and ClbQ and are thus responsive to the colibactin biosynthetic process.

We detected a ClbQ-dependent effect on colibactin expression. Whereas expression of the colibactin determinant at the transcript and protein levels was only weakly affected (Fig. 6; see also Fig. S6D and E), the level of colibactin production, as assessed by the amount of DNA damage and the concentration of the precolibactin cleavage product C14-Asn, was significantly reduced (Fig. 8C). This finding suggests that colibactin production responds to the availability of metabolites, intermediates, or end products of the colibactin biosynthetic process. Both deletion and overexpression of *clbQ* in E. coli M1/5 resulted in reduced levels of γ-H2AX upon bacterial infection of HeLa cells as well as in significant reduction of C14-Asn levels (Fig. 8B and C). This indicates that interference with the biosynthetic flow mediated by increased unloading of intermediates as well as by clogging of the colibactin biosynthesis pipeline reduces the efficacy of colibactin production. Our results therefore corroborate the *in vitro* observation that ClbQ facilitates an additional unloading of colibactin synthesis intermediates as previously reported (26). Whereas the absence of ClbQ results in stalled biosynthesis and reduced mature colibactin levels, increased *clbQ* expression may enhance the release of intermediates from the polyketide assembly line and thus also scale down release of the final colibactin product(s).

Our transcriptome and proteome analyses of the *clbQ* deletion and overexpressing mutants suggest the existence of a regulatory element affecting *clbS* expression located within *clbQ*. While the impact of *clbQ* deletion or overexpression on *clbP* transcript levels can be explained by read mapping to the partially overlapping genes *clbP* and *clbQ*, increased *clbS* expression may result from the presence of a promoter region within *clbQ* which may be affected upon *clbQ* deletion or overexpression (Fig. S6D). Experimental confirmation of transcriptional start sites within the colibactin determinant will be a key future goal to understand in detail the regulation of colibactin expression.

## MATERIALS AND METHODS

### Genome sequencing, assembly, annotation, and gene content analysis.

The genome of E. coli M1/5 was sequenced by combining PacBio and Illumina sequencing technologies. For details on the genome sequencing method, see Text S1 in the supplemental material. Genome assembly was performed with the RS_HGAP_Assembly.3 protocol included in SMRT Portal version 2.2.0. For error correction of PacBio HGAP assembly, Illumina short reads were mapped to the assembled chromosome and plasmid sequences using the Burrows-Wheeler Aligner (BWA) (64). A final quality score of QV60 was confirmed using the RS_BridgeMapper.1 protocol. Automated genome annotation was carried out using PGAP (65). Identification of plasmids, serotypes, and acquired resistance genes was performed with the Web-based tools PlasmidFinder (v1.3) (66), SerotypeFinder (v1.1) (67), and ResFinder (v2.1) (68), respectively. We used a stringent identity threshold of 95% to determine plasmids based on replicon sequences. To examine serotypes and acquired resistance genes, sequence identity levels of 85% and 90% were used, respectively. The length requirement was set to a minimum of 60% sequence coverage for both serotyping and identification of resistance genes. For the determination of virulence factors (VFs), we used the E. coli VF collection (v0.1), which comprises 12 distinct VF groups containing 1,154 deduced protein sequences of virulence-associated genes (69).

10.1128/mSphere.00591-20.1 TEXT S1 Supplemental methods. Download Text S1, PDF file, 0.2 MB. Copyright © 2020 Wallenstein et al. 2020 Wallenstein et al. This content is distributed under the terms of the Creative Commons Attribution 4.0 International license.

### Bacterial strains, plasmids, genetic manipulations, and media.

Information about the strains and plasmids used in this study is provided in Table 2. All E. coli M1/5 mutants generated and used in this study are based on streptomycin-resistant mutant strain E. coli M1/5 *rpsL*K42R (51). For the sake of simplicity, we use the shorter description “M1/5” instead of “M1/5 *rpsL*K42R” in all corresponding mutant designations. Bacterial cultivation was usually performed in lysogeny broth (LB) (10 g liter^−1^ tryptone, 5 g liter^−1^ yeast extract, 5 g liter^−1^ sodium chloride) with shaking at 37°C. If necessary, antibiotics were used at the following concentrations: ampicillin, 100 μg ml^−1^; chloramphenicol, 15 μg ml^−1^ and 25 μg ml^−1^ for low-copy-number and medium-copy-number resistance cassettes, respectively; kanamycin, 50 μg ml^−1^. l-Arabinose was used at a concentration of 3% (wt/vol) to induce *clbR* expression from pBAD-*clbR*. Agar plates were prepared by adding 16 g liter^−1^ agar.

**TABLE 2:** E. coli strains and plasmids used in this study

Strain or plasmid	Genotype and/or characteristics^*a*^	Reference or source
E. coli strains		
DH5α	F^−^ *endA1 hsdR17 supE44 thi-1 recA1 gyrA96 relA1* Δ(*argF*-*lacZYA*) *U169* (Φ60Δ*lacZ* M15λ^−^)	77
Rosetta (DE3)	E. coli strain B; F^−^ *ompT gal dcm lon*? *hsdSB*(r_B_^−^m_B_^−^) *λ*(DE3 [*lacI lacUV5-T7p07 ind1 sam7 nin5*]) [*malB*+]K-12(λ^S^)	Novagen
One Shot pSLC-242	F^c^ *mcrA* Δ(*mrr-hsdRMS*-*mcrBC*) Φ80*lacZ*ΔM15 Δ *lacX74 recA1 araD139* Δ(*araleu*)7697 *galU* *galK rpsL* (Sm^r^) *endA1 nupG*	AddGene/ ThermoFisher
SY327*λpir*	λ(*lac pro*) *argE* (Am) *rif nalA recA56* (λ*pir*)	78
MG1655	K-12 F^−^ λ *ilvG rfb*-*50 rph*-*1*	79
M1/5	Fecal isolate of a healthy individual; *pks*^+^, HPI^+^	This study
Nissle 1917	Fecal isolate of a healthy individual; *pks*^+^, HPI^+^	80
IHE3034	Newborn meningitis E. coli isolate; *pks*^+^, HPI^+^	81
SP15	Newborn meningitis E. coli isolate; *pks*^+^, HPI^+^	82
UTI89	Uropathogenic E. coli isolate; *pks*^+^, HPI^+^	83
Nissle 1917 λ-*attB*::5VNTR- *clbR*p-*lux*	Nissle 1917 *clbR* promoter region containing 5 VNTRs fused with *luxABCDE* integrated into the λ-*attB* site	This study
IHE3034 λ-*attB*::5VNTR- *clbR*p-*lux*	IHE3034 *clbR* promoter region containing 5 VNTRs fused with *luxABCDE* integrated into the λ-*attB* site	This study
SP15 λ-*attB*::5VNTR-*clbR*p-*lux*	SP15 *clbR* promoter region containing 5 VNTRs fused with *luxABCDE* integrated into the λ-*attB* site	This study
UTI89 λ-*attB*::5VNTR-*clbR*p-*lux*	UTI89 *clbR* promoter region containing 5 VNTRs fused with *luxABCDE* integrated into the λ-*attB* site	This study
M1/5 *rpsL*K42R	M1/5 carrying a *rpsL*K42R mutation; Sm^r^	51
M1/5 *rpsL*K42R 19VNTR	M1/5 *rpsL*K42R VNTR region adjusted via scarless mutagenesis to 19 repeats	This study
M1/5 *rpsL*K42R 5VNTR	M1/5 *rpsL*K42R VNTR region adjusted via scarless mutagenesis to 5 repeats	This study
M1/5 *rpsL*K42R Δ*clbQ*	M1/5 *rpsL*K42R Δ*clbQ*::FRT	This study
M1/5 *rpsL*K42R Δ*clbR*	M1/5 *rpsL*K42R Δ*clbRA*::*clbA*-FRT	This study
M1/5 *rpsL*K42R λ-*attB*::FRT	FRT site integrated at λ-*attB* site	This study
M1/5 λ-*attB*::20VNTR-*clbR*p-*lux*	M1/5 *clb*R promoter region containing 20 VNTRs fused with *luxABCDE* integrated into the λ-*attB* site	This study
M1/5 λ-*attB*::5VNTR-*clbR*p-*lux*	M1/5 *clbR* promoter region containing 5 VNTRs fused with *luxABCDE* integrated into the λ-*attB* site	This study

Plasmids		
pASK75	Template for *tetA* promoter cloning	84
pBAD24	Amp^r^; *araC*; *araBAD*p	85
pBAD-*clbR*	For l-arabinose-inducible expression of *clbR* from pBAD24	This study
pBAD24-*clbQ*-*rrnB*t	Template for cloning	This study
pBAD24-*tetA*p-*clbQ*-*rrnB*t	For constitutive expression of *clbQ*	This study
pBAD24-*tetA*p-*clbR*-*rrnB*t	For constitutive expression of *clbR*	This study
pBR322	Template for *rrnB* terminator cloning	86
pCP20	Temperature-sensitive origin of replication, encodes Flp recombinase; Amp^r^, Cm^r^	73
pEX-K4-*tetA*p-*clbR*-*rrnB*t	Contains synthetic *tetA*p-*clbR*-*rrnB*t insert for *clbR* expression	This study
pFuseA-*npt*	pGP704 derivative for chromosomal integration via a FRT sequence; Kan^r^; *oriR6K*; *luxABCDE*	33
pFuseA-*npt*-20VNTR-*clbR*p-*lux*	pFuseA-*npt* derivative carrying 20 VNTRs upstream of a *clbR* promoter-*luxABCDE* fusion; Kan^r^; *oriR6K*	This study
pFuseA-*npt*-5VNTR-*clbR*p-*lux*	pFuseA-*npt* derivative carrying 5 VNTRs upstream of a *clbR* promoter-*luxABCDE* fusion; Kan^r^; *oriR6K*	This study
pGEM-T Easy	TA cloning vector	Promega
pGEM-T Easy-*tetA*p-*clbR*	pGEM-T Easy derivative carrying a *tetA*p-*clbR*-*rrnB*t fusion for inducible expression of *clbR*; Amp^r^	This study
pKD3	Template plasmid for amplification of the FRT-flanked chloramphenicol resistance cassette; FRT-*cat*-FRT; Amp^r^, Cm^r^	71
pKD3-Δ*clbR*1	pKD3 derivative in which the FRT site upstream of *cat* has been replaced by a sequence containing the *clbR* upstream region and the *clbA* gene; *clbR*p::*clbA*- *cat-*FRT; Amp^r^, Cm^r^	This study
pKD4	Template plasmid for amplification of the FRT-flanked kanamycin resistance cassette; Amp^r^, Kan^r^	71
pKD4-'*clbA*	pKD3 derivative with an insertion of the last 524 bp of the *clbA* gene upstream of the FRT-flanked *npt* cassette; '*clbA*-FRT*-npt-*FRT; Amp^r^, Kan^r^	This study
pKD46	Helper plasmid for l-arabinose-inducible expression of λ-Red recombinase (*araC araB*p- *γ-β-exo*); Amp^r^	71
pSLC-242	Template plasmid *cat* cassette for positive selection and *relE* toxin gene under the control of rhamnose inducible promoter (*rhaB*p) for negative selection; Cm^r^	72
pTXB1_*clbR*	pTXB1 with *clbR* gene of E. coli Nissle 1917, for IPTG-inducible expression of a ClbR intein/chitin binding domain fusion for purification; Amp^r^	This study
pUC57-Insert_pFuseA-*npt*_ *clbR*_20VNTR	Contains synthetic insert with 20-repeat VNTR-*clbR*p-*lux* fusion	This study

a Amp^r^, ampicillin resistance; Cm^r^, chloramphenicol resistance; IPTG, isopropyl-β-d-thiogalactopyranoside; Kan^r^, kanamycin resistance; Sm^r^, streptomycin resistance; HPI, high-pathogenicity island.

For growth experiments, the following media were used: M9 medium (70) either with or without 1 g liter^−1^ casein hydrolysate (CAS) (12 g liter^−1^ disodium hydrogen phosphate, 3 g liter^−1^ potassium dihydrogen phosphate, 2 g liter^−1^ glucose, 1 g liter^−1^ ammonium chloride, 0.46 g liter^−1^ sodium chloride, 0.24 g liter^−1^ magnesium sulfate, 0.011 g liter^−1^ calcium chloride, 0.2 mg liter^−1^ thiamine hydrochloride), terrific broth (TB) (70) (12 g liter^−1^ tryptone, 24 g liter^−1^ yeast extract, 5 g liter^−1^ glycerol, 2.31 g liter^−1^ monopotassium phosphate, 12.54 g liter^−1^ dipotassium phosphate), brain heart infusion (BHI) (Fluka, St. Gallen, Switzerland), Todd Hewitt broth (THB) (Oxoid, Wesel, Germany), interaction medium (IM) (Dulbecco’s modified Eagle’s medium [DMEM; Thermo Fisher Scientific, Wesel, Germany] [high glucose, HEPES], supplemented with 1× nonessential amino acids, 2 mM l-alanyl-l-glutamine, 5% [vol/vol] fetal calf serum [FCS]).

For genetic modifications of the bacterial chromosome, the bacteriophage Lambda Red recombinase-dependent approach was used (71), partially refined for scarless mutagenesis (72) or followed by an integrated FRT (FLP recombination target) site/FLP-recombinase-dependent step to generate luciferase-reporter strains (73). The construction of plasmids and mutants is described in detail in Text S1. Oligonucleotides used in this study are given in Table S3 in the supplemental material.

10.1128/mSphere.00591-20.1rev CORRECTED TABLE S3 Oligonucleotides used in this study. Download Table S3, PDF file, 0.1 MB. Copyright © 2020 Wallenstein et al. 2020 Wallenstein et al. This content is distributed under the terms of the Creative Commons Attribution 4.0 International license.

10.1128/mSphere.00591-20.10 ORIGINAL TABLE S3 Oligonucleotides used in this study. Download Table S3, PDF file, 0.1 MB. Copyright © 2020 Wallenstein et al. 2020 Wallenstein et al. This content is distributed under the terms of the Creative Commons Attribution 4.0 International license.

### ClbR purification.

The ClbR protein was heterologously expressed as a ClbR-intein-chitin binding domain fusion using a NEB impact system (New England Biolabs). This allowed chitin affinity chromatography and subsequent thiol-mediated self-cleavage of the intein during elution, yielding tag-less ClbR protein. Expression and purification of ClbR are explained in detail in Text S1.

### Electrophoretic mobility shift assays (EMSA).

Specific interactions of ClbR with DNA were detected using a digoxigenin (DIG) gel shift kit (second generation; Roche Diagnostics, Mannheim, Germany). Regions of interest containing a potential ClbR binding site were amplified via PCR and subjected to DIG labeling. Labeled probes were incubated with rising concentrations of ClbR and poly[d(I·C)] as a nonspecific competitor probing for specific DNA-ClbR interactions. Bound and unbound probes were separated by native polyacrylamide gel electrophoresis followed by a DNA blotting and were then detected via chemiluminescence according to the manufacturer’s protocol. For details, see Text S1.

### Growth-dependent reporter gene assays.

To measure promoter activity via the use of a luminescence reporter (33), we generated reporter fusions by cloning the native *clbB*-to-*clbR* intergenic region with a VNTR region comprising either 5 or 20 repeats into the *attB* locus of E. coli strain M1/5, thereby replacing *clbR* with *luxABCDE*. Luciferase expression in the resulting reporter strains was under the control of the *clbR* promoter. Strains containing the reporter fusion were inoculated 1:100 from overnight cultures in 150 μl in 96-well flat-bottom white polystyrol plates (Greiner Bio-One, Frickenhausen, Germany). OD_600_ and luminescence levels were measured for 23 h in a Tecan Infinite 200 reader (Tecan Group Ltd., Männedorf, Switzerland) (37°C, shaking for 10 min with amplitude setting 2, luminescence integration time 1 s, in 15-min intervals).

### Colibactin cytotoxicity assays.

HeLa cell infection assays were used to analyze the cytotoxic effect of colibactin on mammalian cells via demonstration of megalocytosis and DNA damage (1). The protocols are described in detail in Text S1.

### RNA sequencing.

Bacterial cultures were grown as described for the luminescence assays until an OD_595_ of 0.4 was reached. After pooling of biological replicates, 0.125 volumes of an ethanol-phenol mix (95%:5%) were added and the suspension was incubated 5 min on ice before the bacterial cells were harvested by centrifugation. After the pellet was frozen at –80°C, the cells were thawed and treated with lysozyme by resuspending the pellet in 35 μl Tris-EDTA (TE) buffer that included 85 mg ml^−1^ lysozyme and incubating the samples for 10 min at room temperature. The sample was subjected to vortex mixing every minute for 10 s. Total RNA was extracted by the use of TRIzol (Invitrogen, Karlsruhe, Germany) according to the manufacturer’s protocol. After DNase treatment and PCR-based quality control, the quality of the RNA samples was further assessed by RNA electrophoresis using a 2200 TapeStation system (Agilent). Strand-specific cDNA libraries processed with and without terminator exonuclease (TEX) treatment to enrich primary transcripts were prepared and sequenced (Illumina NextSeq 500, 1× 75-bp single reads) by Vertis Biotechnologie AG (Freising, Germany). Obtained sequencing data were processed using BWA (64) for mapping of transcripts and ReadXplorer 2 (74) for visualization and utilization of differential gene expression results by the use of DESeq2 (75). Differential RNA-seq, i.e., the comparison of results obtained from samples treated with TEX to those obtained from samples left untreated, allowed detection of primary transcripts and determination transcription start sites (76).

### Whole-protein content analysis of RNA sequencing samples.

An aliquot of the bacterial cultures grown for RNA sequencing was also harvested for the analysis of differential protein expression by mass spectrometry. A detailed description of the mass spectrometry-based expression analysis can be found in Text S1.

### Quantification of colibactin intermediate N-myristoyl-d-asparagine.

We compared results representing the ability of E. coli strains to produce colibactin upon growth in M9 medium supplemented with Casamino Acids by quantifying the precolibactin cleavage product N-myristoyl-d-asparagine (C14-Asn) (36, 37). Details are provided in Text S1.

### Statistical analysis.

Statistical analyses were performed using GraphPad Prism software (version 6.0). The figures show mean values with standard deviations (STDEV.P). Unpaired *t* tests were used as indicated. A *P* value of <0.05 was considered statistically significant and is indicated by one asterisk (*). A *P* value of <0.01 is denoted by two asterisks (**), a *P* value of <0.001 by three asterisks (***), and a *P* value of <0.0001 by four asterisks (****).

### Data availability.

The complete genome sequence of E. coli M1/5 (the chromosome and plasmids pM1-5_30 and pM1-5_120) has been deposited at NCBI GenBank under accession numbers CP053296 to CP053298. All RNA-seq data files are available from the Gene Expression Omnibus database (accession no. GSE143807).
