# Erratum for Wallenstein et al., “ClbR Is the Key Transcriptional Activator of Colibactin Gene Expression in Escherichia coli”

**DOI:** 10.1128/mSphere.00727-20

**Published:** 2020-07-29

**Authors:** Alexander Wallenstein, Nadine Rehm, Marina Brinkmann, Martina Selle, Nadège Bossuet-Greif, Daniel Sauer, Boyke Bunk, Cathrin Spröer, Haleluya Tesfaye Wami, Stefan Homburg, Rudolf von Bünau, Simone König, Jean-Philippe Nougayrède, Jörg Overmann, Eric Oswald, Rolf Müller, Ulrich Dobrindt

**Affiliations:** a Institute of Hygiene, University of Münster, Münster, Germany; b Interdisciplinary Center for Clinical Research, Medical Faculty, University of Münster, Münster, Germany; c Institute of Molecular Infection Biology, University of Würzburg, Würzburg, Germany; d IRSD, Université de Toulouse, INSERM, INRA, ENVT, UPS, Toulouse, France; e Department of Microbial Natural Products, Helmholtz Institute for Pharmaceutical Research Saarland, Helmholtz Center for Infection Research, Saarland University, Saarbrücken, Germany; f Leibniz Institute DSMZ—German Collection of Microorganisms and Cell Cultures, Braunschweig, Germany; g DZIF—German Center for Infection Research, Hannover-Braunschweig Partner Site, Braunschweig, Germany; h Pharma Zentrale GmbH, Herdecke, Germany; i Core Unit Proteomics, Interdisciplinary Center for Clinical Research, Medical Faculty, University of Münster, Münster, Germany

## ERRATUM

Volume 5, no. 4, e00591-20, 2020, https://doi.org/10.1128/mSphere.00591-20. Table S3: in the original version of this table, the nucleotide sequences given for primers 1 and 2 were incorrect. A corrected version of Table S3 now appears with the article.

