# Supplementary figures and images for: ClbR Is the Key Transcriptional Activator of Colibactin Gene Expression in Escherichia coli
Source: mSphere. 2020 Jul 15;5(4):e00591-20. doi: 10.1128/mSphere.00591-20 (PMC7364221; doi:10.1128/mSphere.00591-20)

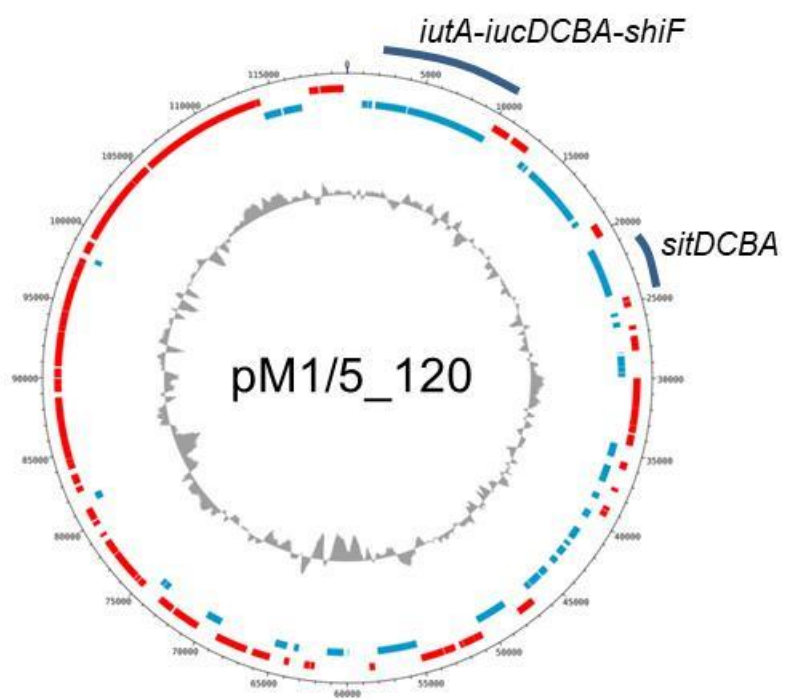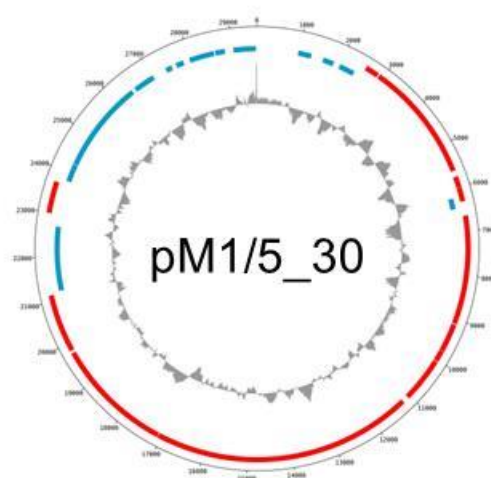

Supplement: FIG S1 [file mSphere.00591-20-sf001.pdf]

**A**

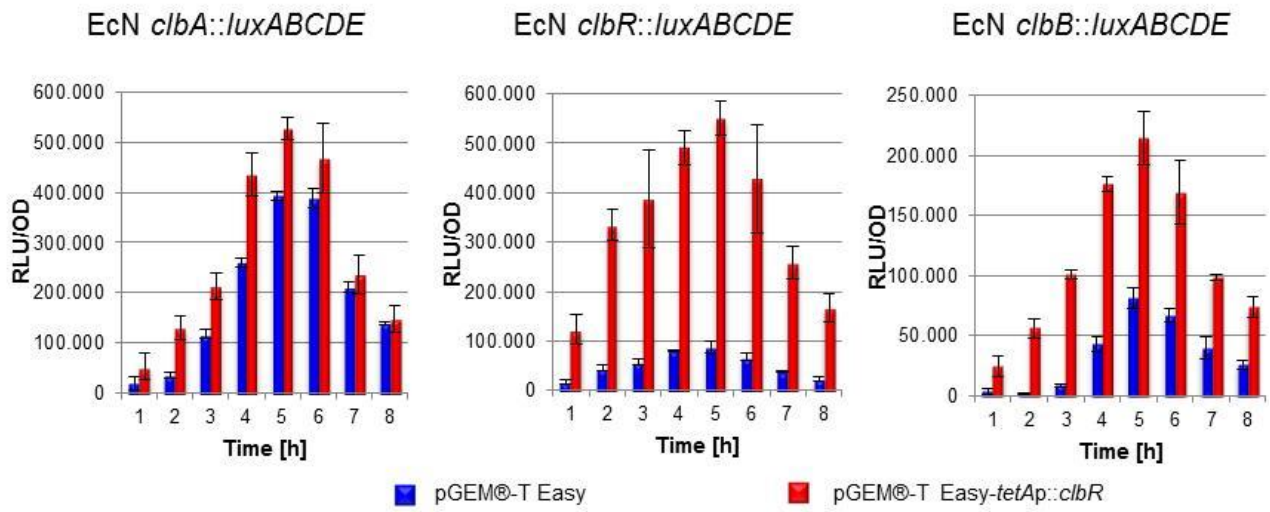

**B**

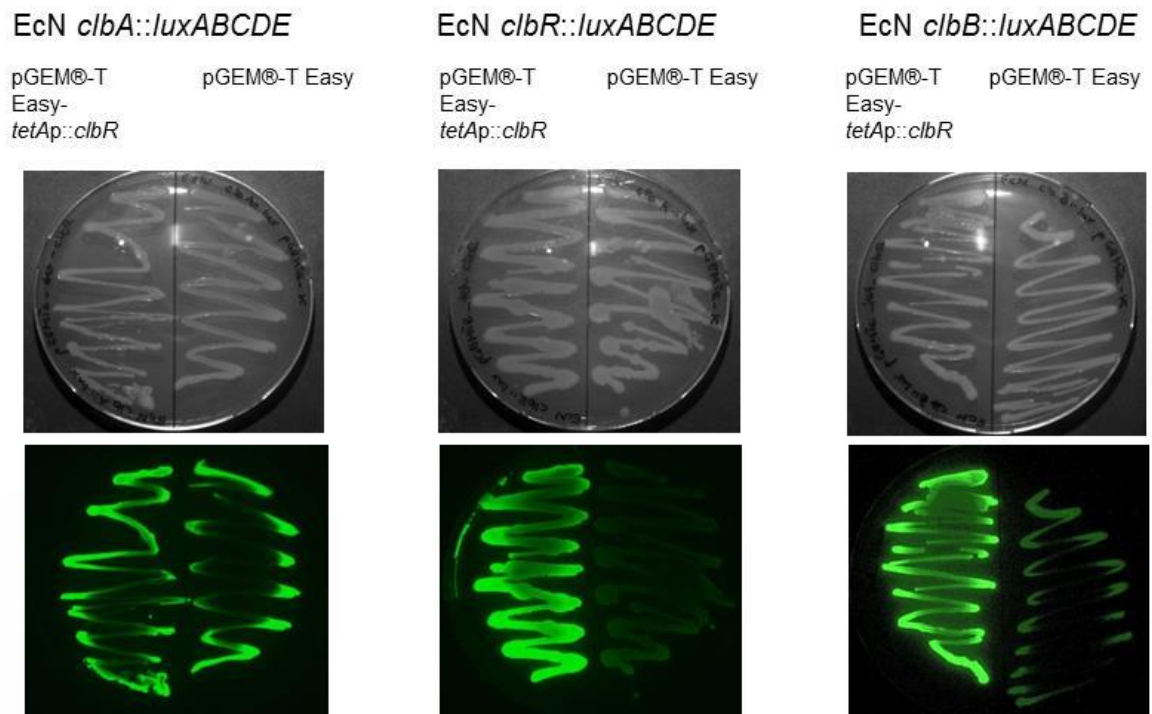

Supplement: FIG S3 [file mSphere.00591-20-sf003.pdf]

**A**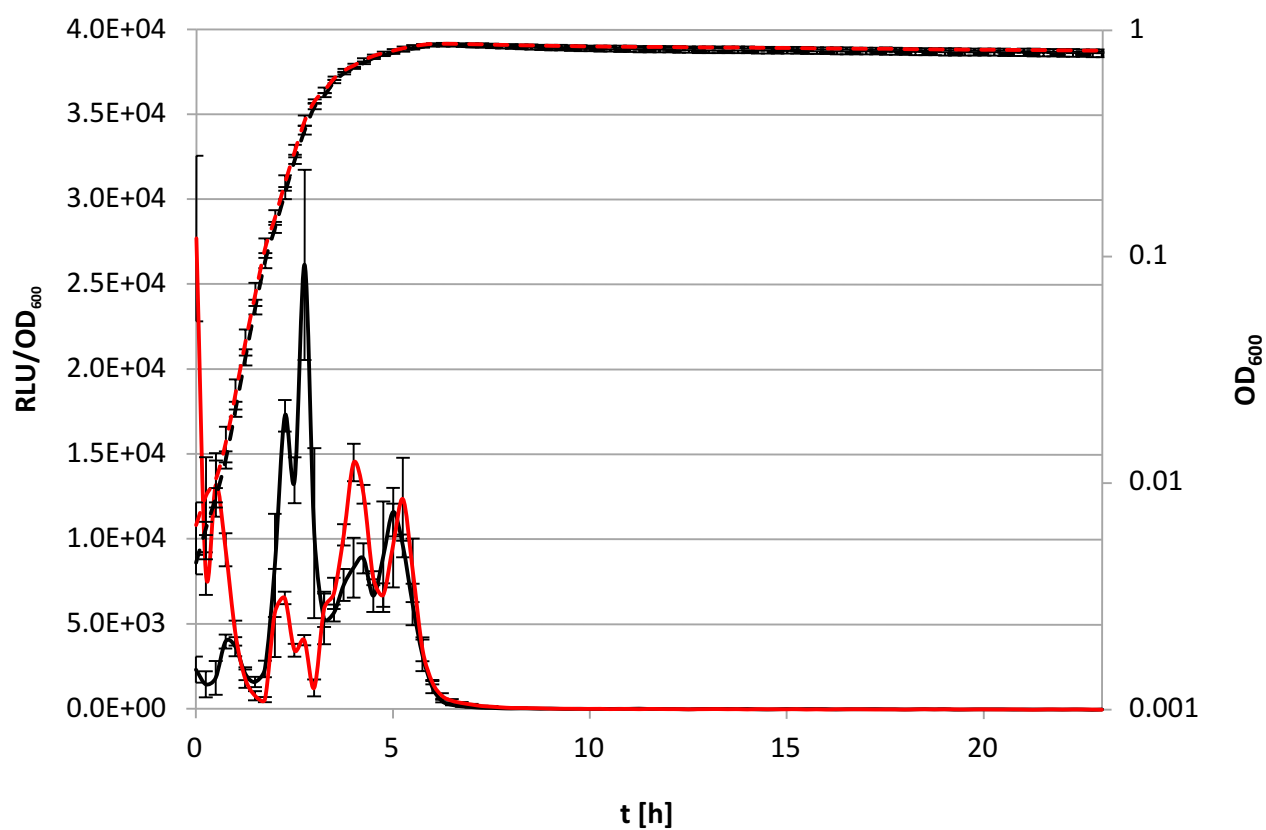**B**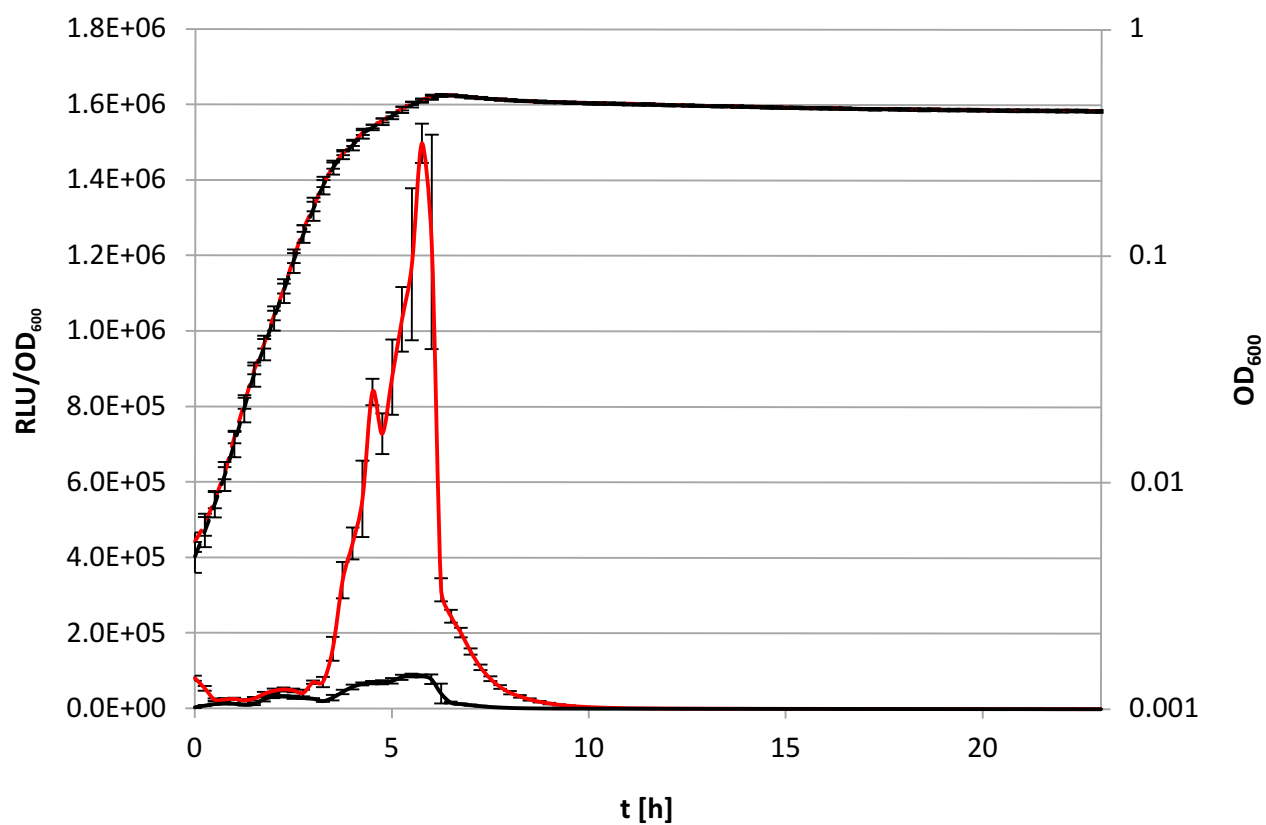

Supplement: FIG S4 [file mSphere.00591-20-sf004.pdf]

**A**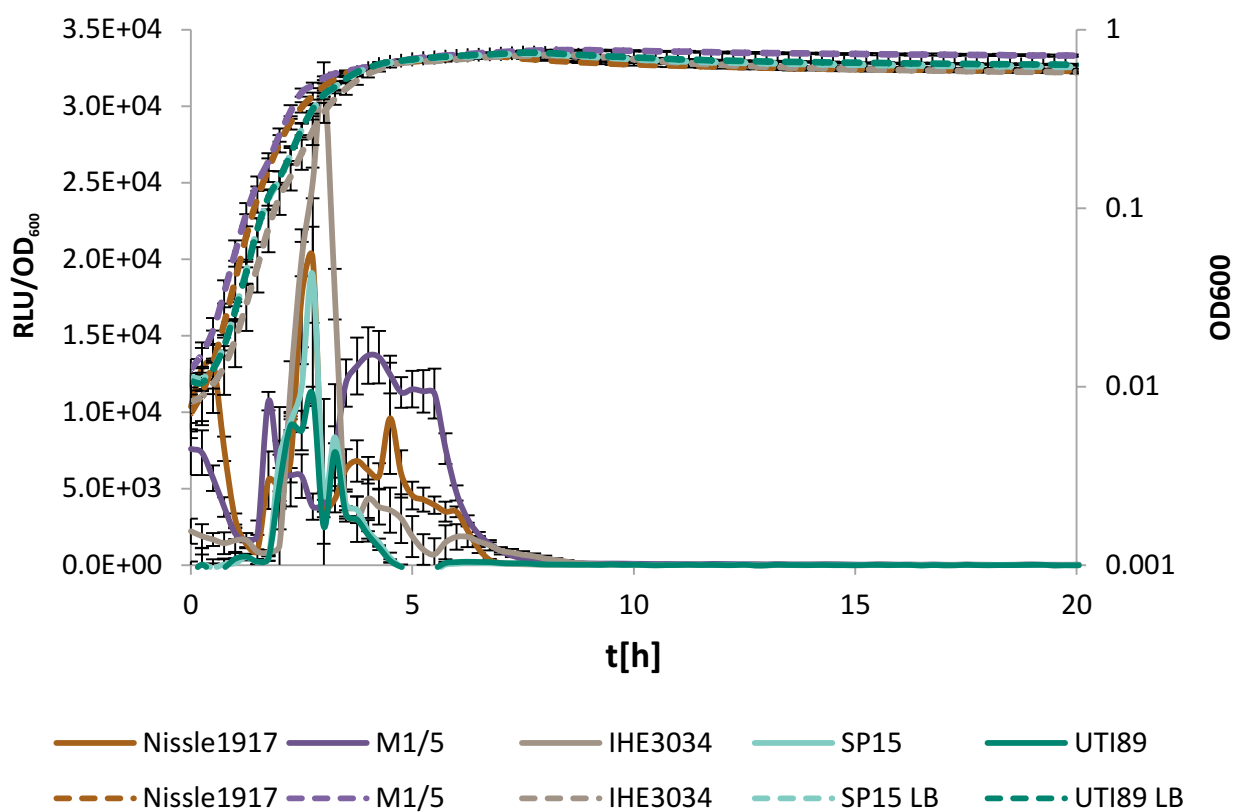**B**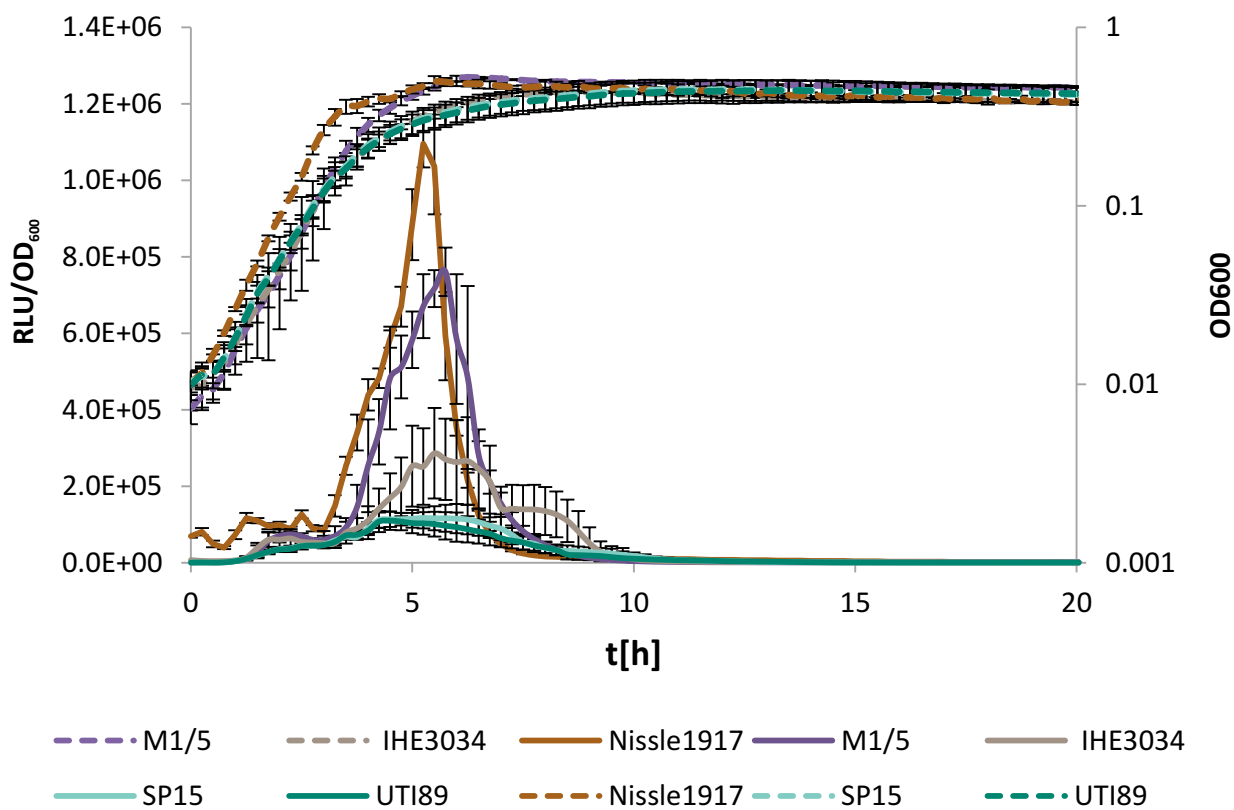

Supplement: FIG S5 [file mSphere.00591-20-sf005.pdf]

**A**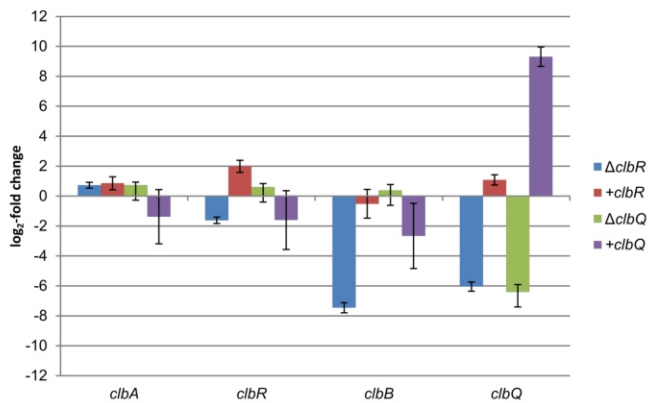**B**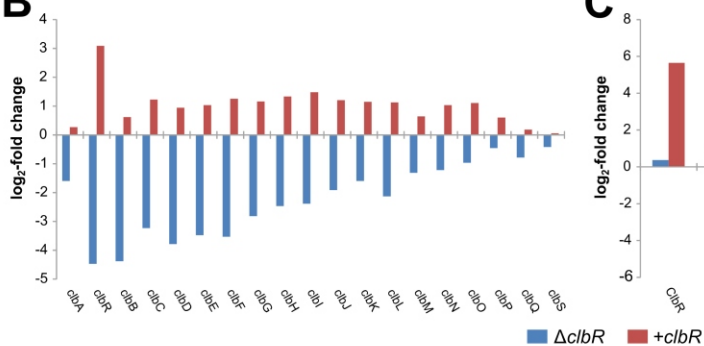**C**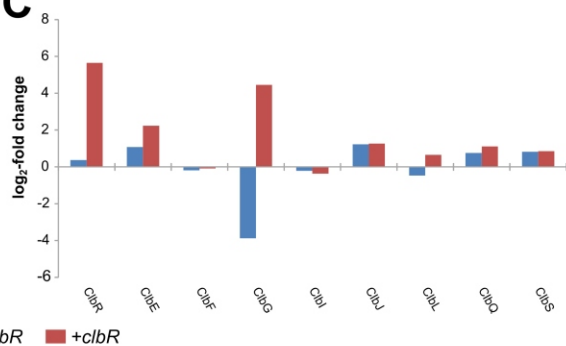**D**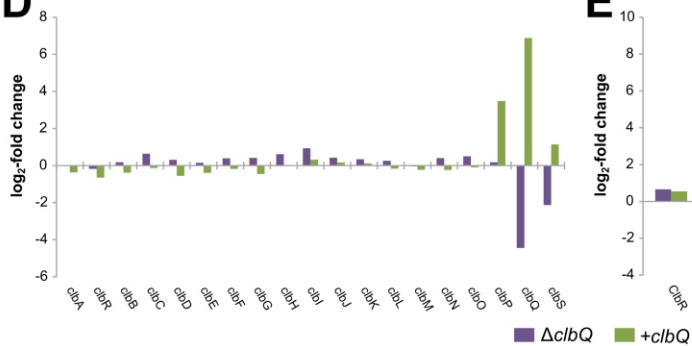**E**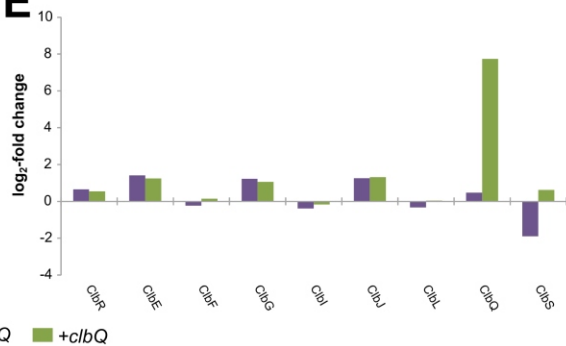

Supplement: FIG S6 [file mSphere.00591-20-sf006.pdf]
