# Supplementary material for: ClbR Is the Key Transcriptional Activator of Colibactin Gene Expression in Escherichia coli
Source: mSphere. 2020 Jul 15;5(4):e00591-20. doi: 10.1128/mSphere.00591-20 (PMC7364221; doi:10.1128/mSphere.00591-20)
Supplement: TABLE S1 [file mSphere.00591-20-st001.pdf]

Table S1: Fitness-related determinants of fecal isolate *E. coli* M1/5

Category	Gene identifier	Funtion
**Adhesins**	HLK60_20945 – HLK60_20910	Type 1 fimbriae (*fimEAICDFGH*)
	HLK_60_01445 - HLK_60_01475	Auf fimbriae (*aufABCDEFG*)
	HLK_60_03095 - HLK_60_03110	CFA/I-like fimbriae (*cfaABCE*)
	HLK_60_18795 - HLK_60_18820	Ecp / Mat fimbriae (*yagZYXWV*)
	HLK_60_19730 - HLK_60_19760	Yad fimbriae (*yadCKLM*, *htrE*, *ecpD*, *yadN*)
	HLK_60_08350 - HLK_60_08365	Yeh fimbriae (*yehABCD*)
	HLK_60_07240 - HLK_60_07270	Yfc fimbriae (*yfcVUTSRQPO*)
	HLK_60_12065 - HLK_60_12085	Yde fimbriae (*ydeQRST*)
	HLK_60_15355 - HLK_60_15385	Curli fimbriae (*csgCABDEFG*)
**T2SS**	HLK_60_01920 - HLK_60_01985<br>HLK_60_03820 - HLK_60_03885	General secretion pathway (*gspOMLKJIHGFEDCA; yghJ, pppA, gspCDEFGHIJKLM, yghG*)
**T5SS**	HLK_60_04040	Autotransporter adhesion antigen 43 (Flu)
	HLK_60_18670<br>HLK_60_12535<br>HLK_60_07750<br>HLK_60_00525	Autotransporter adhesins UpaB, UpaH, UpaI, UpaJ
	HLK_60_04220	Serine autotransporter protease Sat
	HLK_60_18850	Vacuolating autotransporter toxin Vat
	HLK_60_25200	Putative serine autotransporter protease
**T6SS**	HLK_60_05060 - HLK_60_05170	T6SS-2 subtype i2
	HLK_60_19205 - HLK_60_19340<br>HLK_60_12265<br>HLK_60_12300	T6SS-1 subtype i1 and additional Vgr-family proteins
**Iron uptake systems**	HLK_60_04320 - HLK_60_04255	Aerobactin biosynthesis – ShiF major facilitator gene cluster I
	HLK_60_25025 - HLK_60_25050	Aerobactin biosynthesis – ShiF major facilitator gene cluster II
	HLK_60_09280 - HLK_60_09330	Yersiniabactin biosynthesis
	HLK_60_04160	Bifunctional enterobactin receptor/adhesin protein Iha
	HLK_60_01045 - HLK_60_01080	Hemin uptake (*hmuV, chuUYXWTAD*)
	HLK_60_15455 - HLK_60_15445	Ferrous low pH iron transport (*efeUOB*)
	HLK_60_17405 - HLK_60_17425<br>HLK_60_17455 - HLK_60_17480<br>HLK_60_16435	Enterobactin biosynthesis/transport (*entHABEC; entD, fepA, fes, ybdZ, entF, fepE; fiu*)
	HLK_60_21035 - HLK_60_21065	Ferric citrate transport (*fecIRABCDE*)
	HLK_60_01580 - HLK_60_01575	ferrous iron transport (*feoAB*)

	HLK_60_19670 - HLK_60_19685<br>HLK_60_03465 - HLK_60_03490	Ferrichrome iron transport (*fhuBDCA; fitABCDER*)
	HLK_60_14430 - HLK_60_14445	Ferrous iron/manganese transport system I (*sitABCD*)
	HLK_60_25115 - HLK_60_25130	Ferrous iron/manganese transport system II (*sitABCD*)
	HLK_60_09070	Heme receptor Hma
**Other**	HLK_60_09115 - HLK_60_09205	Colibactin biosynthesis (*clbARBCDEFGHIJKLMNOPQS*)
	HLK_60_03890 - HLK_60_03955	K5 capsule (*kpsMT, kfiABCD, kpsSCUDEF*)
	HLK_60_15725	Outer membrane protein A
	HLK_60_17620	Outer membrane protease OmpT, omptin
	HLK_60_09840 - HLK_60_09990;<br>HLK_60_10125 - HLK_60_10195;<br>HLK_60_15160 - HLK_60_15225	Flag-1 flagellar system
	HLK_60_18935 - HLK_60_19150	Flag-2 flagellar system
	HLK_60_08130	Colicin 1 receptor CirA
	HLK_60_07365	Colicin V production protein
	HLK_60_25375	Colicin 1A immunity protein
	HLK_60_25335	Complement resistance protein TraT
