# Supplementary material for: ClbR Is the Key Transcriptional Activator of Colibactin Gene Expression in Escherichia coli
Source: mSphere. 2020 Jul 15;5(4):e00591-20. doi: 10.1128/mSphere.00591-20 (PMC7364221; doi:10.1128/mSphere.00591-20)

A

```
ClbR    MGGNMDKFKEKNPLSLRERQVLRMLAQGDEYSQISHNLNISINTVKFHVKNIKHKIQARNTNHAIHIANR-NEII-   74
GerE    --MKEKEFQSKPLLTKREREVFELLVQDKTTKEIASELFISEKTVRNHISNAMQKLGVKGRSQAVVELLRMGELEL   74
          : .:*:.*  *: ***:*:.:*.*..  .:*: :* ** :**: *:.*  :*: .:..:*:    * .*:
```

B

```
1        10        20        30        40        50        60        70
MGGNMDKFKEKNPLSLRERQVLRMLAQGDEYSQISHNLNISINTVKFHVKNIKHKIQARNTNHAIHIANRNEII
---------------HHHHHHHHHHHH---HHHHHHHH---HHHHHHHHHHHHHH-----HHHHHHHHHH----
```

C

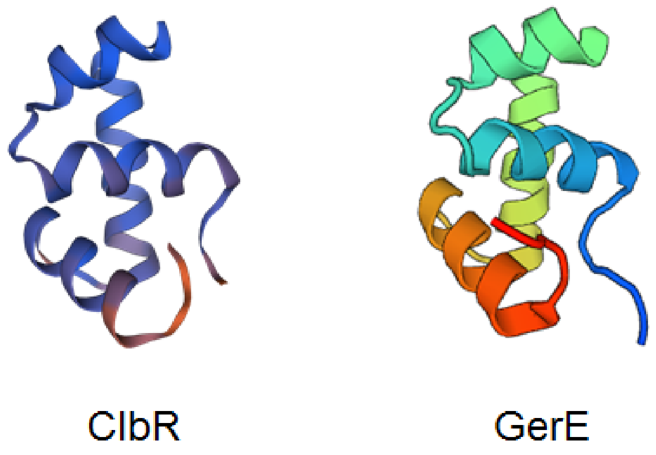

Supplement: FIG S2 [file mSphere.00591-20-sf002.pdf]
