# Supplementary material for: ClbR Is the Key Transcriptional Activator of Colibactin Gene Expression in Escherichia coli
Source: mSphere. 2020 Jul 15;5(4):e00591-20. doi: 10.1128/mSphere.00591-20 (PMC7364221; doi:10.1128/mSphere.00591-20)
Supplement: TABLE S2 [file mSphere.00591-20-st002.pdf]

Table S2: Differentially expressed genes and proteins in *E. coli* M1/5 in response to altered *clbR* or *clbQ* expression.

Identifier	Annotation	M1/5 *rpsL*K42R Δ*clbR* (pBAD24)	M1/5 *rpsL*K42R (pBAD24-*tetA*p-*clbR*-*rrnB*t)	M1/5 *rpsL*K42R Δ*clbQ* (pBAD24)	M1/5 *rpsL*K42R (pBAD24-*tetA*p-*clbQ*-*rrnB*t)
De-regulated Genes					
**HLK60_20145**	*araC*	0.125	-1.900	0.876	-3.206
**HLK60_06095**	*aroF*	1.597	2.000	1.980	1.694
**HLK60_09125**	*clbB*	-4.381	0.613	0.179	-0.391
**HLK60_09130**	*clbC*	-3.236	1.222	0.639	-0.149
**HLK60_09135**	*clbD*	-3.794	0.935	0.304	-0.566
**HLK60_09140**	*clbE*	-3.482	1.029	0.150	-0.410
**HLK60_09145**	*clbF*	-3.536	1.245	0.396	-0.184
**HLK60_09150**	*clbG*	-2.820	1.157	0.410	-0.461
**HLK60_09155**	*clbH*	-2.470	1.330	0.611	0.022
**HLK60_09160**	*clbI*	-2.388	1.474	0.930	0.304
**HLK60_09175**	*clbL*	-2.133	1.120	0.256	-0.182
**HLK60_09195**	*clbP*	-0.455	0.600	0.182	3.466
**HLK60_09200**	*clbQ*	-0.792	0.178	-4.445	6.861
**HLK60_09120**	*clbR*	-4.473	3.085	-0.174	-0.662
**HLK60_09205**	*clbS*	-0.424	0.046	-2.137	1.125
**HLK60_22730**	*dgkA*	-0.049	-2.100	-0.699	-0.392
**HLK60_20655**	*fhuF*	-0.177	-2.769	-0.062	-0.354
**HLK60_19145**	*flhB*	-1.848	0.544	-1.957	3.345
**HLK60_09980**	*fliA*	-1.405	-3.264	-1.515	-1.463
**HLK60_08760**	*hisA*	-1.506	-2.991	-3.538	-2.878
**HLK60_08770**	*hisB*	-1.853	-2.605	-2.543	-2.594
**HLK60_08775**	*hisC*	-2.286	-2.889	-2.304	-2.634
**HLK60_08780**	*hisD*	-2.135	-2.553	-1.211	-2.359
**HLK60_08755**	*hisF*	-1.005	-3.016	-3.389	-2.481
**HLK60_08765**	*hisH*	-2.293	-2.974	-3.403	-2.856
**HLK60_00110**	*yidH*	-0.920	-0.078	-2.252	0.110
**HLK60_00250**	hypothetical protein	0.917	2.129	1.485	2.345
**HLK60_03660**	*yghW*	-0.533	-0.815	-1.149	-2.216
**HLK60_08465**	*yegR*	-1.653	-2.382	-1.062	-1.581
**HLK60_08750**	*hisI*	-0.347	-2.550	-3.164	-2.230
**HLK60_08905**	hypothetical protein	1.765	2.451	0.807	2.045
**HLK60_09335**	integrase	1.180	-0.456	1.070	2.345
**HLK60_09590**	hypothetical protein	-1.990	-2.041	-0.778	-1.918
**HLK60_12140**	hypothetical protein	-0.458	-2.246	-0.217	-0.638
**HLK60_12255**	hypothetical protein	-0.405	0.544	0.485	2.252
**HLK60_13680**	hypothetical protein	-1.493	-0.843	-1.795	-2.743
**HLK60_14510**	hypothetical protein	-0.331	-1.382	-0.762	-2.166
**HLK60_18780**	*ykgM*	1.456	2.984	2.147	0.900
**HLK60_20130**	*yabI*	-0.688	-3.558	0.265	-3.813

Identifier	Annotation	M1/5 *rpsL*K42R Δ*clbR* (pBAD24)	M1/5 *rpsL*K42R (pBAD24-*tetA*p-*clbR*-*rrnB*t)	M1/5 *rpsL*K42R Δ*clbQ* (pBAD24)	M1/5 *rpsL*K42R (pBAD24-*tetA*p-*clbQ*-*rrnB*t)
**HLK60_25335**	hypothetical protein	1.568	2.340	1.611	0.264
**HLK60_25435**	*ssb*	0.917	1.351	0.485	2.593
**HLK60_25465**	hypothetical protein	-0.727	1.222	0.164	2.023
**HLK60_00085**	*ibpB*	-0.790	0.589	0.390	3.683
**HLK60_22775**	*malG*	0.425	1.074	-0.363	2.082
**HLK60_10305**	*mepM*	1.203	2.056	1.289	0.765
**HLK60_04320**	*papA*	-2.106	-0.456	0.107	-0.655
**HLK60_17685**	*purK*	-4.106	-1.157	-0.630	-0.449
**HLK60_13375**	*pyrF*	1.710	1.766	2.070	2.618
**HLK60_18785**	*rpmJ*	1.748	3.274	2.382	0.809
**HLK60_11510**	*sodB*	-0.183	-2.041	-0.136	-0.070
**HLK60_25530**	*traV*	1.080	1.922	0.485	2.193
**HLK60_13485**	*trpCF*	1.116	1.702	1.458	2.061
**HLK60_13480**	*trpD*	1.019	2.134	1.441	2.116
**HLK60_13475**	*trpE*	1.187	2.600	1.388	2.050
**HLK60_06100**	*tyrA*	1.744	1.950	2.100	1.849
**HLK60_21480**	*ulaE*	0.180	0.544	0.070	2.667
**HLK60_09915**	*ybcL*	-0.121	-2.105	-0.231	-0.554
**HLK60_01370**	*yhhY*	1.595	2.470	1.864	1.345
**HLK60_23835**	*yihQ*	0.917	0.544	0.485	2.345
**HLK60_23805**	*yihV*	0.180	2.351	0.485	0.667
**HLK60_09740**	*zinT*	1.634	2.912	1.975	0.671
**HLK60_10300**	*znuA*	1.248	2.001	1.265	0.731
De-regulated Proteins					
**HLK60_00030**	YidA	1.817	2.217	1.802	1.675
**HLK60_00080**	IbpA	0.045	0.714	0.191	3.588
**HLK60_00085**	IbpB	-0.173	0.347	0.162	3.662
**HLK60_00300**	Gmk	1.616	2.359	2.263	1.789
**HLK60_01035**	HdeB	-1.287	-2.219	-1.823	-1.876
**HLK60_01060**	HutX	2.35	2.756	2.337	2.222
**HLK60_01135**	UspA	1.49	2.199	2.004	1.707
**HLK60_01340**	YhhA	-1.806	-2.383	-1.754	-2.431
**HLK60_01690**	Rpe	1.955	2.169	2.037	1.832
**HLK60_01695**	Gph	2.33	2.621	2.132	2.079
**HLK60_01700**	TrpS	3.851	4.138	3.99	3.645
**HLK60_01810**	hypothetical protein	5.648	5.234	5.653	4.43
**HLK60_01820**	YheU	-1.433	-2.246	-1.056	-2.146
**HLK60_02040**	RpsQ	4.543	5.069	5.228	4.665
**HLK60_02405**	Rng	2.314	2.364	2.116	2.341
**HLK60_02475**	ZapE	8.609	11.652	7.697	9.869
**HLK60_02490**	SspA	1.598	1.69	2.442	1.825
**HLK60_02570**	RapZ	3.638	3.381	3.666	3.451
**HLK60_02575**	PtsN	5.47	5.808	4.822	5.84

Identifier	Annotation	M1/5 *rpsL*K42R Δ*clbR* (pBAD24)	M1/5 *rpsL*K42R (pBAD24-*tetA*p-*clbR*-*rrnB*t)	M1/5 *rpsL*K42R Δ*clbQ* (pBAD24)	M1/5 *rpsL*K42R (pBAD24-*tetA*p-*clbQ*-*rrnB*t)
**HLK60_02620**	MlaF	11.053	12.572	12.334	11.065
**HLK60_02770**	RpsO	1.622	1.626	2.075	1.645
**HLK60_03010**	TdcF	2.212	2.929	3.084	2.666
**HLK60_03040**	YhaJ	-1.326	-2.085	-1.399	-1.588
**HLK60_03195**	EbgC	2.693	3.888	4.429	3.227
**HLK60_04605**	YgfI	14.106	14.365	14.276	13.784
**HLK60_04925**	LysA	2.231	2.32	2.076	2.129
**HLK60_05115**	hypothetical protein	1.573	1.719	2.178	1.713
**HLK60_05260**	FucO	10.81	12.507	12.176	10.744
**HLK60_05285**	QueF	3.057	2.584	3.092	2.858
**HLK60_05525**	IspF	-2.102	-3.419	-2	-2.884
**HLK60_05550**	RpoS	10.935	12.901	10.814	10.503
**HLK60_05895**	ProV	1.638	2.015	2.148	1.742
**HLK60_06030**	NadK	11.572	11.514	11.479	11.678
**HLK60_06255**	RseB	-1.63	-2.494	-2.745	-1.912
**HLK60_06610**	PurM	3.233	3.226	3.272	3.118
**HLK60_06715**	AegA	-0.243	-0.112	-0.066	2.569
**HLK60_06725**	YpfG	4.894	5.559	5.483	4.546
**HLK60_06890**	CysA	4.103	4.487	4.317	4.203
**HLK60_06935**	ZipA	3.148	3.338	3.751	2.705
**HLK60_06940**	LigA	1.959	2.21	1.647	1.882
**HLK60_07430**	YfcE	14.227	14.598	14.197	13.131
**HLK60_07465**	HxpA	4.955	5.67	5.667	5.109
**HLK60_07480**	AlaA	2.782	2.678	1.968	2.838
**HLK60_07660**	NudI	2.146	0.654	2.118	1.506
**HLK60_07710**	GlpB	2.287	1.841	2.746	1.819
**HLK60_07825**	RcsB	2.401	2.783	2.508	2.383
**HLK60_07975**	YejK	2.288	2.535	2.79	2.148
**HLK60_08555**	Dcd	4.343	4.348	4.6	4.173
**HLK60_08680**	RfbB	1.841	2.363	1.83	1.955
**HLK60_08685**	RfbD	2.749	3.613	3.046	2.884
**HLK60_08690**	RfbA	3.06	3.319	3.222	3.351
**HLK60_08725**	CpsG	2.278	2.52	2.391	2.574
**HLK60_08745**	WzzB	4.449	4.784	4.434	4.263
**HLK60_08765**	HisH	1.398	2.75	1.233	1.492
**HLK60_08835**	YeeX	2.242	2.01	2.224	2.113
**HLK60_08925**	hypothetical protein	11.598	10.787	11.291	10.634
**HLK60_09120**	ClbR	0.374	5.656	0.655	0.545
**HLK60_09130**	ClbC	nd	13.256	11.4	10.511
**HLK60_09135**	ClbD	1.084	2.232	1.411	1.241
**HLK60_09145**	ClbF	-3.876	4.455	1.227	1.061
**HLK60_09200**	ClbQ	0.757	1.11	0.472	7.738

**Identifier**	**Annotation**	**M1/5 *rpsL*K42R Δ*clbR* +pBAD24**	**M1/5 *rpsL*K42R pBAD24-*tetA*p-*clbR*-*rrnB*t**	**M1/5 *rpsL*K42R Δ*clbQ* +pBAD24**	**M1/5 *rpsL*K42R pBAD24-*tetA*p-*clbQ*-*rrnB*t**
**HLK60_09285**	YbtE	3.522	3.591	3.51	2.837
**HLK60_09820**	YedP	12.047	11.734	11.861	10.658
**HLK60_10090**	YecI	4.126	4.065	4.298	3.872
**HLK60_10115**	OtsA	2.567	2.519	2.217	2.17
**HLK60_10690**	MsrB	8.078	8.115	7.919	8.271
**HLK60_11185**	hypothetical protein	1.67	2.006	1.831	1.813
**HLK60_11190**	FtsH4	-2.291	-2.411	-1.626	-1.144
**HLK60_11355**	MenI	2.24	2.495	2.815	2.442
**HLK60_11375**	SufC	3.396	4.011	3.655	2.927
**HLK60_11410**	FumD	-0.335	-0.328	0.499	-2.232
**HLK60_11675**	YdgJ	7.288	10.847	11.969	10.315
**HLK60_11720**	UidC	10.84	11.973	11.238	11.385
**HLK60_11930**	Dcp	2.071	2.277	2.177	1.799
**HLK60_12230**	hypothetical protein	8.692	8.962	8.656	8.42
**HLK60_12660**	hypothetical protein	-2.785	-4.59	nd	nd
**HLK60_13010**	hypothetical protein	1.693	2.208	2.181	1.974
**HLK60_13445**	CobO	3.674	3.224	3.869	3.74
**HLK60_13525**	hypothetical protein	11.474	12.082	12.141	11.2
**HLK60_13820**	hypothetical protein	4.456	4.565	4.234	4.31
**HLK60_14080**	YchN	3.466	3.675	3.613	3.222
**HLK60_14185**	DhaL	2.301	1.09	1.413	1.945
**HLK60_14355**	YmgD	2.145	2.512	1.576	1.252
**HLK60_14565**	NudJ	7.085	7.131	6.926	7.231
**HLK60_14630**	hypothetical protein	3.488	2.766	3.082	2.86
**HLK60_14990**	LolE	3.822	4.016	4.398	3.981
**HLK60_15295**	YceI	12.259	12.835	12.257	12.489
**HLK60_15395**	YcdY	2.854	3.001	3.451	0.704
**HLK60_15515**	YmdF	-2.259	-2.759	-3.518	-1.847
**HLK60_15595**	CspG	1.453	2.707	2.363	2.214
**HLK60_15685**	YccU	4.484	4.664	4.356	4.042
**HLK60_15850**	LdtD	4.767	5.611	4.606	4.984
**HLK60_16545**	MoaC	-2.629	-1.936	-2.128	-2.429
**HLK60_16635**	YbhA	2.588	2.853	2.571	2.823
**HLK60_16685**	GalM	1.92	2.741	2.51	2.469
**HLK60_16785**	YbgC	3.036	3.148	2.939	2.856
**HLK60_16835**	SdhB	0.785	0.929	0.931	2.841
**HLK60_16880**	PxpA	10.21	11.775	11.038	11.13
**HLK60_17470**	Fes	2.885	3.503	3.403	2.793
**HLK60_17680**	PurE	1.891	2.073	1.839	1.922
**HLK60_17900**	Gsk	3.196	2.607	2.9	3.027
**HLK60_18100**	ClpP	2.091	2.395	2.099	2.186
**HLK60_18175**	YajL	1.541	2.321	1.929	1.357
**HLK60_18385**	IraP	1.924	2.456	2.129	1.855

Identifier	Annotation	M1/5 *rpsL*K42R Δ*clbR* +pBAD24	M1/5 *rpsL*K42R pBAD24-*tetA*p-*clbR*-*rrnB*t	M1/5 *rpsL*K42R Δ*clbQ* +pBAD24	M1/5 *rpsL*K42R pBAD24-*tetA*p-*clbQ*-*rrnB*t
**HLK60_18425**	IprA	2.609	2.725	2.567	2.575
**HLK60_18570**	YahO	-4.35	-4.754	-5.681	-5.62
**HLK60_19435**	MetN	3.334	3.631	3.549	3.585
**HLK60_19650**	ErpA	-1.264	-1.563	-1.321	-2.124
**HLK60_19720**	PcnB	2.967	3.251	2.622	2.547
**HLK60_19765**	PanB	3.497	3.941	3.46	3.381
**HLK60_19870**	PdhR	11.001	8.99	12.011	10.076
**HLK60_20005**	MurG	4.409	4.446	5.217	4.637
**HLK60_20015**	MurD	2.109	2.007	2.331	2.055
**HLK60_20025**	MurF	4.864	3.312	5.564	3.33
**HLK60_20030**	MurE	0.692	0.964	0.686	2.505
**HLK60_20035**	FtsI	1.869	2.471	2.207	1.864
**HLK60_20040**	FtsL	10.182	12.306	11.802	9.358
**HLK60_20270**	CaiA	7.572	13.873	10.616	9.894
**HLK60_20440**	ThrB	2.024	2.552	3.308	2.407
**HLK60_20900**	UxuA	2.899	3.218	2.84	3.029
**HLK60_21180**	PepA	1.804	2.124	1.875	1.681
**HLK60_21205**	RraB	0.064	0.268	3.284	-1.199
**HLK60_21210**	ArgF	2.306	2.727	2.516	2.384
**HLK60_21595**	Rnr	0.071	0.112	0.071	3.467
**HLK60_21690**	RsgA	2.852	1.684	2.872	2.217
**HLK60_21725**	FrdB	-1.89	-2.515	-4.851	-2.047
**HLK60_21745**	Blc	2.207	2.148	2.323	1.975
**HLK60_22150**	hypothetical protein	2.179	2.741	2.194	2.134
**HLK60_22190**	YdiA	3.551	4.157	3.074	8.014
**HLK60_22725**	LexA	13.849	14.026	13.907	13.262
**HLK60_22820**	YjbD	-0.895	-1.444	-2.315	-1.284
**HLK60_22980**	YjaG	2.883	3.341	2.497	2.758
**HLK60_23140**	BtuB	2.61	1.811	3.157	1.758
**HLK60_23200**	ArgC	1.619	1.85	2.415	1.975
**HLK60_23240**	FrwB	-1.661	-2.314	-1.435	-1.977
**HLK60_023370**	ZapB	3.578	3.653	3.95	3.288
**HLK60_23380**	GlpK	3.387	2.948	3.178	3.279
**HLK60_24260**	hypothetical protein	2.167	2.583	1.92	2.499
**HLK60_24305**	CyaY	11.889	11.993	13.599	11.855
**HLK60_24315**	HemC	8.077	9.203	8.858	8.571
**HLK60_24495**	IlvG	1.911	2.18	1.748	2.052
**HLK60_24675**	AtpC	2.573	2.544	2.574	2.445
**HLK60_24750**	YieH	6.191	6.227	6.784	6.297
**HLK60_25190**	hypothetical protein	2.534	2.591	1.468	2.424
**HLK60_24910**	VirB11	3.213	3.441	2.696	3.019

De-regulated genes and proteins are listed with a fold-change relative to the wild type with a base mean ≥10 and a $\log_2$-fold change ≤-2 or ≥+2. Indicated are changes in gene/protein expression relative to wild type strain M1/5 *rpsL*K42R (pBAD24).
