# Supplementary material for: ClbR Is the Key Transcriptional Activator of Colibactin Gene Expression in Escherichia coli
Source: mSphere. 2020 Jul 15;5(4):e00591-20. doi: 10.1128/mSphere.00591-20 (PMC7364221; doi:10.1128/mSphere.00591-20)
Supplement: TEXT S1 [file mSphere.00591-20-s0001.pdf]

Cloning

Strains and plasmids were partially sequenced for quality control. PCR reactions were done with Q5-Polymerase (New England Biolabs) according to the manufacturer's protocol. All *E. coli* M1/5 mutants generated and used in this study are based on the streptomycin-resistant mutant strain *E. coli* M1/5 *rpsL*K42R (1). For the sake of simplicity we used in the manuscript the shorter description "M1/5" instead of "M1/5 *rpsL*K42R" in all corresponding mutant designations. Primers used for cloning are listed in Supplementary Table S1.

Plasmids

pFuseA-*npt*-5VNTR-*clbR*p-*lux*

This plasmid was created following a restriction free cloning approach (2). With primers 1+2 an integration primer was amplified based on M1/5 *rpsL*K42R. After fusing the desired sequence to pFuseA-*npt* with the integration primer by PCR, *Dpn*I digestion of template and religation of the target vector, an intermediate plasmid was created. To avoid sequencing of the whole plasmid, a portion comprising the insert was cut and inserted into pFuseA-*npt* using *BstE*II and *Rsr*II. With this method we were able to obtain a DNA fragment containing 5VNTR and cloned it into pFuseA-*npt* via *BstE*II and *Rsr*II.

pFuseA-*npt*-20VNTR-*clbR*p-*lux*

A synthetic insert from pUC57-Insert_pFuseA-*npt_clbR*_20VNTR, generated by BioCat, was subcloned into pFuseA-*npt* via *BstE*II and *Rsr*II.

pBAD24-*clbQ-rrnB*t

The insert was amplified with primers 3 and 4 using *E. coli* M1/5 genomic DNA as a template. This way, restriction sites were fused with the desired sequence. The insert was cloned into pBAD24 via *EcoR*I and *Xba*I.

pBAD24-*tetA*p-*clbR-rrnB*t

A synthetic insert (*tetA*p-*clbR-rrnB*t) provided in pEX-K4-*tetA*p-*clbR-rrnB*t (generated by Eurofins) was subcloned via *Pci*I and *Pfl*MI into pBAD24.

pBAD24-*tetA*p-*clbQ-rrnB*t

The insert was generated by a fusion PCR using primers 5 and 6 and the following fragments: The *tetA* promoter fragment was generated with primers 7 and 8 from pASK75. The *clbQ-rrnB*t fragment was generated with primers 9 and 6 from pBAD24-*clbQ-rrnB*. The fusion fragment was cloned into pBAD24 via *Pci*I and *Pfl*MI.

pKD3-Δ*clbR*1

This plasmid was constructed as a template plasmid for amplification of the *clbR* upstream region and of *clbA* directly fused to the chloramphenicol resistance (*cat*) cassette. In a first step, a PCR product comprising the *clbR* upstream region was generated with primers 10 and 11 from template *E. coli* M1/5 *rpsL*K42R. A second PCR product was generated by amplification of the *clbA* gene from the same template using primers 12 and 13. Both PCR products were fused using primers 10 and 13. The resulting PCR product was cut with *Afe*II and *Swa*I and then ligated with pKD3 digested with A*fe*II and *Swa*I leading to plasmid pKD3-Δ*clbR*1.

pKD4-'*clbA*

This plasmid served as a template plasmid for amplification of a partial *clbA* sequence followed by the *npt* kanamycin resistance cassette flanked by FRT sites. For this purpose, an overlap PCR product was generated. One PCR product, comprising 524 bp of the end of the *clbA* gene, was generated with primers 14 and 15 and *E. coli* M1/5 *rpsL*K42R as a template. A second PCR product comprising the FRT site and a part of the *npt* gene was amplified from pKD4 using primers 16 and 17. The two PCR products were then fused using primers 14 and 18 in a way that the subcloned part of the *clbA* gene preceded the FRT site upstream of the *npt* cassette. The resulting PCR product was cut with *Bmg*BI and *Bgl*II and ligated with pKD4, which had been digested before with the same enzymes, thus yielding pKD4-'*clbA*.

pTXB1_*clbR*

After amplifying *clbR* from *E. coli* strain Nissle 1917 with primers 18 and 19, the generated fragment was cloned into pGEM©-T Easy via T/A-cloning. The resulting plasmid and pTXB1 were digested with *Sap*I and *Nde*I and ligated, resulting in pTXB1_*clbR*.

pBAD-*clbR*

The *clbR* coding sequence was amplified with primers 20 and 21. The terminator of *rrnB* was amplified from pBR322 using the primers 22 and 23. Both fragments were purified and used together as starting material for a fusion PCR with primers pBAD-clbR-up and rrnB-lp. The resulting PCR product was ligated into pBAD24.

pGEM®-T Easy-*tetA*p-*clbR*

First, the *tetA* promoter of plasmid pASK75 was amplified with the primers 24 and 25. This way, a sequence displaying homology to the 5'-end of *clbR* was added to the 3'-end of the *tetA* promoter region. In addition, *clbR* was amplified together with the terminator of *rrnB* from pBAD24_*clbR* using the primers 26 and 27. Both fragments were purified and used together

as starting material for a fusion PCR with primers 28 and 29. The resulting PCR product was ligated into pGEM®-T Easy.

Chromosomal modification

E. coli strains were generated based on principles described before (3-5). Primers used for genetic modifications were listed in Supplementary Table S1.

***E. coli* strains modified for λ*attB*::FRT**

E. coli strains where transformed with pKD46 and subsequently transformed with a PCR product generated from pKD3 with primers 30 and 31, which enables insertion of a chloramphenicol resistance cassette (*cat*) into the *attB* locus by recombineering. A chloramphenicol-resistant clone was then transformed with pCP20 to remove the *cat* cassette by FLP recombinase-mediate recombination between the two FRT sites.

E. coli* strains λ*attB*::5VNTR-*clbR*p-*lux* and *E. coli* M1/5 *rpsL*K42R λ*attB*::20VNTR-*clbR*p-*lux

Strains carrying reporter gene fusions where created by the same strategy. *E. coli* strains modified to λ*attB*::FRT were transformed with pCP20. The pCP20-encoded FLP recombinase was used to insert pFuseA-*npt*-5VNTR-*clbR*p-*lux* or pFuseA-npt-20VNTR-*clbR*p-*lux* into the FRT-site.

E. coli* strain M1/5 *rpsL*K42R λ*attB*::*cat-frr*p-*lux

To generate a control strain with λ-red recombinase through pKD46, *E. coli* strains λ*attB*::5VNTR-*clbR*p-*lux* was used as acceptor. The insert was generated by recombinant PCR. The *frr*p region was amplified with primer pair 32+33 from chromosomal DNA. A product with overlap of the *frr*p site to *luxA* was generated with primers 34+35 based on pFuseA-*npt*-5VNTR-*clbR*p-*lux*, the *cat*-cassette was obtained with primer pair 31+36 from pKD3. First the *frr*P region was fused with the *luxA*-fragment and simultaneously an overlap towards *cat*-cassette was added by PCR with primer pair 34+37. The final insert combined the *cat*-cassette with *frr*p-*luxA* by using previous products and 31+34.

***E. coli* M1/5 *rpsL*K42R 5VNTR, *E. coli* M1/5 *rpsL*K42R 19VNTR**

All three strains were created based on the method described by Khetrapal and co-workers (4). Two λ-red recombinase-mediated recombination events were necessary, catalyzed by pKD46. First a selection cassette containing a positive selection marker (*cat* resistance cassette), together with a negative selection marker (the toxic gene *relE* under control of the rhamnose-dependent promoter of *rhaB*), were inserted upstream of *clbR*. This insert was

generated from pSLC-242 with primers 38 and 39. After positive selection, strains carrying the cassette with both markers were again transformed with pKD46 to replace the cassette by a PCR fragment the desired sequence, which just differed in the VNTR repeat number from the wild type strain M1/5. Those inserts were generated with primers 40 and 41 from *E. coli* strains or plasmids carrying VNTR regions of different size.

E. coli* M1/5 *rpsL*K42R Δ*clbR

The *E. coli* M1/5 *rpsL*K42R Δ*clbR* deletion mutant results from several allelic replacement events. Successful curing of pKD46 was verified by PCR using primers 42 and 43. If required, antibiotic resistance cassettes were removed by transformation with pCP20 encoding the FLP recombinase (5). The *clbR* locus is located directly upstream of the *clbA* gene and the transcriptional organization of this region has not been fully characterized. To delete *clbR* without modifying the immediate upstream sequence, *clbR* and *clbA* had first to be deleted in parallel in *E. coli* strain M1/5 *rpsL*K42R, before the *clbA* coding sequence was re-inserted at its original locus. First, a kanamycin resistance cassette was amplified from pKD4 with primers 44 and 45 and used for replacement of the *clbRA* locus in *E. coli* strain M1/5 (pKD46). In the resulting mutant *E. coli* M1/5 Δ*clbRA* (pKD46) only the *clbR* start codon remained whereas the rest of the *clbR* coding region and the complete *clbA* gene were deleted. This strain was then transformed with a PCR product amplified from pKD3-Δ*clbR*1 with primers 45 and 46. The PCR product comprised 132 bp of the *clbR* upstream region, the full *clbA* gene, a *cat* cassette, an FRT site and 55 nucleotides of the sequence downstream of *clbA*. The large sequence homologous to the upstream region of *clbR* was required to prevent a possible change of the VNTR repeat number located directly upstream of *clbR* during recombination. The PCR product inserted in a way that the kanamycin resistance cassette of *E. coli* M1/5 *rpsL*K42R Δ*clbRA* was not removed, but located downstream of the chloramphenicol resistance gene due to recombination of the homologous FRT site sequences upstream of *npt* and downstream of *cat*. The resulting strain, *E. coli* M1/5 *rpsL*K42R Δ*clbR-cat-npt* lacked the *clbR* gene, but contained the complete *clbA* gene. Transformation of this strain with pCP20 led to removal of the kanamycin resistance cassette resulting in *E. coli* strain M1/5 *rpsL*K42R Δ*clbR-cat*. Since we aimed at a marker-free *clbR* deletion mutant, *E. coli* M1/5 *rpsL*K42R Δ*clbR-cat* (pKD46) was transformed with a PCR product comprising 213 bp of the downstream part of the *clbA* gene, the kanamycin resistance cassette flanked by FRT sites and 55 bp of the sequence downstream of *clbA*. This sequence was amplified from

pKD4-'*clbA* using primers 45 and 47. When the resulting strain *E. coli* M1/5 *rpsL*K42R Δ*clbR-npt* was transformed with pCP20, the *npt* cassette was removed and the final strain, *E. coli* M1/5 Δ*clbR*, was obtained carrying a full *clbR* deletion as well as an FRT site downstream of the *clbA* gene.

ClbR purification

The ClbR protein was synthesized as a ClbR-intein-chitin binding domain fusion using the NEB Impact System (New England Biolabs). For this purpose, *E. coli* strain Rosetta(DE3) (pTXB1_*clbR*) was grown in 50 mL LB containing 100 µg mL^{-1} ampicillin at 37 °C until an OD_{600} of 0.6 was reached. Expression of the fusion protein was induced with 0.2 mM IPTG, and the culture was grown for 20 h at 22 °C. Bacteria were harvested by centrifugation and pellets were resuspended in 11.4 mL column buffer (20 mM HEPES, 500 mM sodium chloride, 1 mM EDTA, cOmplete™ Protease Inhibitor (Roche Diagnostics, Mannheim, Germany), pH 8.5). Cells were disrupted with ceramic beads and vigorous shaking using the Precellys instrument (Ozyme, Montigny le Bretonneux, France). Cell debris and non-disrupted cells were removed by centrifugation; cell extracts were treated with Benzonase (Merck Millipore, Darmstadt, Germany) to degrade nucleic acids and then loaded on a column with 3 mL 50 % chitin matrix (New England Biolabs) previously equilibrated with 20 volumes of column buffer. The matrix containing the ClbR-intein-chitin binding domain fusion protein was washed first with 20 volumes of wash buffer 1 (column buffer containing 0.1 % Triton X-100), then 20 volumes of wash buffer 2 (wash buffer 1 containing 1 M sodium chloride) and subsequently treated with three volumes of cleavage buffer (column buffer containing 50 mM 2-mercaptoethanol). The column was incubated for 40 h at room temperature to allow 2-mercaptoethanol-mediated cleavage of the intein part of the ClbR-intein-chitin binding domain fusion protein. Native ClbR was eluted with 16 times of 0.5 ml of column buffer and samples were analyzed by SDS-PAGE. Fractions containing high amounts of ClbR protein were pooled and concentrated to 0.7 mL using Vivaspin centrifugal concentrators with a molecular weight cut-off of 3 kDa (Sigma-Aldrich, Taufkirchen, Germany). The column buffer was exchanged to TEN buffer (10 mM Tris, 1 mM EDTA, pH 7.5, 100 mM sodium chloride) in a ratio of 1:10,000 by consecutive dilution with TEN buffer and concentration by centrifugation using the Vivaspin device. ClbR concentration was estimated by SDS-PAGE using determined BSA amounts for calibration.

Electrophoretic mobility shift assays (EMSA)

To detect specific interactions of ClbR with DNA a DIG Gel Shift kit (2nd Generation, Roche Diagnostics, Mannheim, Germany) was used. Regions of interest containing a potential interaction site were amplified via PCR (using primers 48 to 67, Supplementary Table S1) and digoxigenin (DIG) labeled. Probes were purified using Quick Spin Sephadex G25 Columns (Roche) and concentrations were determined with the TapeStation 2200 (Agilent Technologies, Waldbronn, Germany). Afterwards labeled probes were incubated in (in 10 µl reaction volume) with rising ClbR concentration (0 nM-150 nM) and poly [d(I-C)] as non-specific competitor probing for specific DNA-ClbR interaction. In addition to the manufacturer's protocol we added 0.1 µg bovine serum albumin (BSA) per reaction to ensure specificity and stability of the interaction (6). Bound and unbound probes were separated by native polyacrylamide gel electrophoresis in 8 %-polyacrylamid-Tris-borate-EDTA (TBE) gels, run for 120 min at 110 V in 0.5 x TBE-buffer, followed by a DNA-blot. We transferred the separated probes on a positively charged nylon membrane (42 min, 300 mA) and crosslinked the DNA by UV irradiation (2 x 1200 units) and further processed according to manufacturer's protocol. Images were obtained and analyzed wit a ChemiDoc XRS+ System (Bio-Rad, Munich, Germany)

Growth dependent reporter gene assays

To measure promoter activity via a luminescence reporter (7), we generated reporter fusions by cloning the native *clbB* to *clbR* intergenic region with a VNTR region comprising either five or 20 repeats into the *attB* locus of *E. coli* strain M1/5, thereby replacing *clbR* with *luxABCDE*. Luciferase expression in the resulting reporter strains is under control of the *clbR* promoter. Strains containing the reporter fusion were inoculated 1:100 from overnight cultures in 150 µl in 96 flat bottom white polystyrene plates (Greiner Bio-One, Frickenhausen, Germany). OD_{595} and luminescence were measured for 23 h in a Tecan Infinite 200 microplate reader (Tecan Group Ltd., Männedorf, Switzerland) (37 °C, shaking 10 min with amplitude 2, luminescence integration time 1s, in 15 min intervals).

To test the impact of iron availability on *clbR* promotor activity, bacteria were grown under the conditions described above for 1h 15min. Then, either ferric chloride, the chelator deferroxamine or water was added up to a final volume of 160 µl. To study the iron dependency of clbR promoter activity, the available iron concentration was either increased

by adding $FeCl_3$ to a concentration of 100 µM or decreased upon the addition of 0.2 µM deferroxamin.

Colibactin cytotoxicity assays

HeLa cells, maintained by serial passage in Eagle's minimum essential medium (EMEM) supplemented with 10 % fetal calf serum (FCS) and non-essential amino acids at 37 °C and 5 % CO_2, were used to demonstrate the cytotoxic effect of colibactin on mammalian cells. Colibactin has been shown to induce double strand breaks, which leads to cell cycle arrest and therefore formation of megalocytotic cells (8). To examine the extent of DNA double strand breaks using serine139-phosphorylated histone H2AX, γ-H2AX, induced by bacteria synthesizing colibactin, 1.25×10^5 HeLa cells were dispersed in a 6-well cell culture plate. For analysis of megalocytosis, a 24-well cell culture plate with 5×10^4 HeLa cells was prepared. The next day, bacterial overnight cultures were used to infect HeLa cells with a multiplicity of infection (MOI) of 100 in interaction medium. Four hours post infection cells were washed four times with 1x Hank´s Balanced Salt Solution (HBSS) (Thermo Fisher Scientific, Wesel, Germany) containing 100µg mL^{-1} gentamycin and further incubated at 37 °C and 5 % CO_2 in DMEM with 10 % FCS, non-essential amino acids and 200 µg mL^{-1} gentamycin.

To visualize megalocytosis, HeLa cells were washed twice with 1x HBSS 48 hours post infection and then used for standard phase contrast microscopy. For cell cycle analysis, nuclear suspensions were made directly from adherent cells with 0.1% sodium citrate, 1% NP-40, 50 µg of propidium iodide/ml, and 250 µg of RNase/ml. Nuclei DNA content data were acquired with a MACSQuant Analyzer 10 (Milteny Biotec) flow cytometer. Flow cytometry data were analyzed using FlowJo 7 software.

For γ-H2AX detection, cells were washed eight hours post infection with ice-cold phosphate buffered saline (137 mM sodium chloride, 2.7 mM potassium chloride, 10.1 mM disodium hydrogen phosphate, 1.8 mM potassium dihydrogen phosphate, pH 7.4) and immediately suspended and lysed with sample buffer (32.9 mM Tris, 15 % (v/v) glycerol, 0.5 % sodium dodecyl sulfate, pH 6.8). Total protein in cell extracts was quantified using BCA reagent (Thermo Fisher Scientific, Wesel, Germany). 4 or 6 µg total protein were first separated by SDS-PAGE using 4-20 % Mini-Protean-TGX gels (Bio-Rad) and then transferred onto a polyvinyl difluoride (PVDF) membrane. For detection of γ-H2AX, a monoclonal rabbit anti-phospho-histone H2AX (Ser139) antibody (Millipore, Darmstadt, Germany) and as secondary antibody a goat anti-rabbit-antibody coupled with horse radish peroxidase (HRP) (Dianova

GmbH, Hamburg, Germany) was used. Alternatively, anti-gammaH2A.X (phospho S139) primary antibody (Abcam, Cambridge, UK) and as secondary antibody the horseradish peroxidase (HRP)-coupled AffiniPure Goat Anti-Rabbit IgG (H+L) (Dianova GmbH, Hamburg, Germany) were applied. As a control, β-actin was detected with anti-beta actin [AC-15] antibody (HRP) (Abcam). As substrate for horseradish peroxidase Bio-Rad Clarity Western ECL-substrate was used (Bio-Rad). Blots were analyzed using the ChemiDoc XRS+ System (Bio-Rad).

Genome sequencing

Genomic DNA of *E. coli* strain M1/5 was isolated using QIAGEN Genomic Tip/100 G Kit (Qiagen, Hilden, Germany) and PureLink Genomic DNA Mini Kit (Life Technologies, USA). For PacBio sequencing, the SMRTbell™ template library was prepared as outlined in the Procedure & Checklist-10 kb Template Preparation and Sequencing. Briefly, for preparation of 10 kb libraries, ~15 μg genomic DNA was sheared using g-tubes™ (Covaris, Woburn, MA, USA). DNA was end repaired and ligated to hairpin adapters using the DNA/Polymerase Binding Kit P4 according to the manufacturer's instructions (Pacific Biosciences). BluePippin™ Size-Selection was performed according to the manufacturer's instructions (Sage Science, Beverly, MA, USA). SMRT sequencing was carried out on the PacBio RS*II* (Pacific Biosciences) on three SMRT Cells taking 180-minute movies. Sequencing yielded 162,570 post-filtered reads with roughly 1.4 gigabase of sequence information.

Furthermore, total genomic DNA was sequenced on a MiSeq system (Illumina, Netherlands). For this purpose, a Nextera XT paired-end library was prepared and sequenced using v2 chemistry. From this 2,781,286 bp paired-end reads of 2x100 bp were available.

RNA sequencing

Bacterial cultures were grown as described for the luminescence assays until they reached an OD_{595} of 0.4. After pooling of biological replicates to a total volume of 3.36 ml, 0.125 volumes of an ethanol-phenol mix (95%:5%) was added, the suspension was incubated 5 min on ice, before the bacterial cells were harvested by centrifugation. After freezing the pellet at -80 °C, the cells were thawed and treated with lysozyme. Total RNA was extracted according to manufacturer's protocol with TRIzol™ (Invitrogen, Karlsruhe, Germany). After DNase treatment and PCR-based quality control, the quality of the RNA samples was further

assessed by RNA electrophoresis using the 2200 TapeStation (Agilent). Strand-specific cDNA libraries with and without Terminator Exonuclease (TEX) treatment to enrich primary transcripts were prepared and sequenced (Illumina NextSeq 500, 1 x 75 bp single reads) by vertis Biotechnologie AG (Freising, Germany). Obtained sequencing data was processed using BWA (9) for mapping of transcripts, ReadXplorer 2 (10) for visualization and utilization of differential gene expression by DESeq 2 (11). Differential RNA-seq, i.e. the comparison of +/-TEX treated samples, allows to detect primary transcripts and determine transcription start sites (12).

Whole protein content analysis of *E. coli* M1/5

From the same batch cultures grown for the RNA-seq experiment biological replicates were first pooled (final volume of 3.36 ml), and then harvested by centrifugation. Cells were lysed in 8 M urea, 100 mM Tris, 2%(w/v) SDS (pH 8.5) using a Labsonic M device (Sartorius, 3 x 10 s, on ice) and subsequently centrifuged (4 °C, 30 min, 20.000 x g). The pooled supernatants were diluted 1:4 with a solution of 8 M urea and 100 mM Tris to reach 0.5 % (w/v) SDS (pH 8). The protein concentration was determined using the Cytoskeleton kit ADV01 and proteins were prepared for mass spectrometry-based expression analysis with Synapt G2 Si coupled to M-Class nanoUPLC (Waters Corp.) as previously described (13). Briefly, proteins were reduced, alkylated, digested using trypsin and injected into the instrument at 250 ng/µl (0.5 and 1 µl injections). Measurements were performed in replicate and data were analysed using Progenesis (Nonlinear Diagnostics) and the genome sequence of *E. coli* M1/5. The resulting normalized abundance values were used to calculate $\log_2$-fold changes for the individual proteins detected in the respective sample versus the reference strain *E. coli* M1/5 (pBAD24).

Cluster and Gene Ontology analysis

The expression profiles of genes and proteins with a $\log_2$-fold change ≥+/-2 in their expression levels were analyzed by hierarchical cluster analysis using Cluster 3.0. The heat maps were generated with Java TreeView (14, 15). Functional enrichment analysis of the groups of genes or proteins with similar expression profiles was performed with STRING db (https://string-db.org/) (16)

Quantification of the precolibiactin cleavage product N-myristoyl-D-asparagine

We compared the ability of *E. coli* strains to produce colibactin by quantifying the precolibactin cleavage product N-myristoyl-D-asparagine (17, 18). This N-terminal prodrug motif is generated by ClbN and then cleaved from the synthesized colibactin molecule by ClbP, thus reflecting total amount of polyketide produced (19). Strains were grown as batch cultures at 37 °C for 24 h in glass tubes with 5 ml M9 medium containing casamino acids and 200 µl of Amberlite™ XAD-16 resin/water slurry. Cells and XAD-16 resins were harvested by centrifugation, resuspended in a final volume of 100 ml acetone and stirred for 1 h. The samples were sedimented and filtered, and after another round of resin incubation and stirring in 100 ml acetone for 1 h, the suspensions were sedimented and filtered a second time. The resulting final 200 ml solvent samples were removed with a rotary evaporator and afterwards resuspended in 1.6 ml methanol. After centrifugation for 10 min with 15,000 rpm at 4 °C, 1.5 ml of the supernatant was dried in a vacuum centrifuge and resuspended in 50 µl methanol. Finally, 30 µl were transferred into a glass vial and measured in 0.5 µl injection volumes. Samples were analyzed by Ultra Performance Liquid Chromatography coupled to High Resolution Mass Spectrometry (UPLC-HRMS) with a Thermo Scientific Ultimate 3000 RS on a Waters Acquity BEH 100*2.1mm 1.7µm 130A column, eluent A: 0.1 % formic acid in ddH_2O, eluent B: 0.1 % formic acid in acetonitrile, at a flow rate of 0.6 ml/min followed by a Bruker Maxis II - 4G (Bruker Daltonics, Bremen, Germany) with 150-2500 m/z and a scan rate of 2Hz. As an internal standard 250 mM cinnarizine was used. Peak areas were analyzed by normalization with the internal standard and OD_{600} of the bacterial culture at the time point of harvesting.

RT-qPCR of prominent *clb* genes

To support our RNA-seq findings, reverse transcriptase- quantitative PCR (RT-qPCR) was conducted (Fig. S8). RNA samples were extracted from three biological replicates, grown shaking, at 37°C in glass tubes containing 5ml M9 medium with casamino acids, as described for RNA sequencing to be used as template. A KAPA SYBR® FAST One-step kit (Kapa Biosystems) was used as following: In 10 µl Reaction volume 1ng/µl template. 0.2 µM of the respective primers (*gapA*, primers 68 and 69; *clbA*, primers 70 and 71; *clbR*, primers 72 and 73; *clbB*, primers 74 and 75; *clbQ*, primers 76 and 77) and according kit reagents were set up. The gene *gapA* was used as a reference. No RT control and no template controls were run

accordingly. Reactions were carried out corresponding to manufacturer's recommendations in a BioRad CFX96/C1000 RT PCR thermal cycler. The PCR program is described below. C_t values were obtained during annealing and extention by BioRad CFX Manager Software and further processed following double delta C_t method described before (20).

**Step**	**Temperature**	**Time**	**Cycles**
cDNA synthesis	42°C	5 min	
Inactivate RT	95°C	5 min	
Denature	95°C	3 s	40x
Anneal/extend	60°C	25 s	
Melting curve	65°C to 95°C. in 0.5°C steps	-	

References

1. Martin P, Marcq I, Magistro G, Penary M, Garcie C, Payros D, Boury M, Olier M, Nougayrède JP, Audebert M, Chalut C, Schubert S, Oswald E. 2013. Interplay between siderophores and colibactin genotoxin biosynthetic pathways in *Escherichia coli*. PLoS Pathog 9:e1003437.
2. Chen GJ, Qiu N, Karrer C, Caspers P, Page MG. 2000. Restriction site-free insertion of PCR products directionally into vectors. Biotechniques 28:498-500, 504-5.
3. Datsenko KA, Wanner BL. 2000. One-step inactivation of chromosomal genes in *Escherichia coli* K-12 using PCR products. Proc Natl Acad Sci U S A 97:6640-5.
4. Khetrapal V, Mehershahi K, Rafee S, Chen S, Lim CL, Chen SL. 2015. A set of powerful negative selection systems for unmodified *Enterobacteriaceae*. Nucleic Acids Res 43:e83.
5. Cherepanov PP, Wackernagel W. 1995. Gene disruption in *Escherichia coli*: TcR and KmR cassettes with the option of Flp-catalyzed excision of the antibiotic-resistance determinant. Gene 158:9-14.
6. Hellman LM, Fried MG. 2007. Electrophoretic mobility shift assay (EMSA) for detecting protein-nucleic acid interactions. Nat Protoc 2:1849-61.
7. Homburg S, Oswald E, Hacker J, Dobrindt U. 2007. Expression analysis of the colibactin gene cluster coding for a novel polyketide in *Escherichia coli*. FEMS Microbiol Lett 275:255-62.
8. Nougayrède JP, Homburg S, Taieb F, Boury M, Brzuszkiewicz E, Gottschalk G, Buchrieser C, Hacker J, Dobrindt U, Oswald E. 2006. *Escherichia coli* induces DNA double-strand breaks in eukaryotic cells. Science 313:848-51.
9. Li H, Durbin R. 2010. Fast and accurate long-read alignment with Burrows-Wheeler transform. Bioinformatics 26:589-95.
10. Hilker R, Stadermann KB, Schwengers O, Anisiforov E, Jaenicke S, Weisshaar B, Zimmermann T, Goesmann A. 2016. ReadXplorer 2-detailed read mapping analysis and visualization from one single source. Bioinformatics 32:3702-3708.
11. Love MI, Huber W, Anders S. 2014. Moderated estimation of fold change and dispersion for RNA-seq data with DESeq2. Genome Biol 15:550.
12. Sharma CM, Hoffmann S, Darfeuille F, Reignier J, Findeiss S, Sittka A, Chabas S, Reiche K, Hackermüller J, Reinhardt R, Stadler PF, Vogel J. 2010. The primary transcriptome of the major human pathogen *Helicobacter pylori*. Nature 464:250-5.
13. König S, Hadrian K, Schlatt S, Wistuba J, Thanos S, Böhm MRR. 2019. Topographic protein profiling of the age-related proteome in the retinal pigment epithelium of *Callithrix jacchus* with respect to macular degeneration. Journal of Proteomics 191:1-15.
14. de Hoon MJ, Imoto S, Nolan J, Miyano S. 2004. Open Source Clustering Software. Bioinformatics 20:1453-4.
15. Eisen MB, Spellman PT, Brown PO, Botstein D. 1998. Cluster analysis and display of genome-wide expression patterns. Proc Natl Acad Sci USA 95:14863-8.
16. von Mering C, Huynen M, Jaeggi D, Schmidt S, Bork P, Snel B. 2003. STRING: a database of predicted functional associations between proteins. Nucl Acids Res 31:258-61.
17. Bian X, Fu J, Plaza A, Herrmann J, Pistorius D, Stewart AF, Zhang Y, Müller R. 2013. *In vivo* evidence for a prodrug activation mechanism during colibactin maturation. Chembiochem 14:1194-7.
18. Bian X, Plaza A, Zhang Y, Müller R. 2015. Two more pieces of the colibactin genotoxin puzzle from. Chem Sci 6:3154-3160.
19. Brotherton CA, Balskus EP. 2013. A prodrug resistance mechanism is involved in colibactin biosynthesis and cytotoxicity. J Am Chem Soc 135:3359-62.
20. Livak KJ, Schmittgen TD. 2001. Analysis of relative gene expression data using real-time quantitative PCR and the 2(-Delta Delta C(T)) Method. Methods 25:402-8.
