# Supplementary material for: ClbR Is the Key Transcriptional Activator of Colibactin Gene Expression in Escherichia coli
Source: mSphere. 2020 Jul 15;5(4):e00591-20. doi: 10.1128/mSphere.00591-20 (PMC7364221; doi:10.1128/mSphere.00591-20)
Supplement: ORIGINAL TABLE S3 [file mSphere.00591-20-st003.pdf]

Table S3: Oligonucleotides used in this study

Primer	Sequence (5`-3`)	Function
1	CGAACTGTTCGCCAGGCTC	Cloning of pFuseA-*npt*-5VNTR-*clbR*p-*lux*
2	AGAAGTTCCTATTCTCTAGAAAGAATAG	Cloning of pFuseA-*npt*-5VNTR-*clbR*p-*lux*
3	GCGCGAATTCACCATGAGTAATATCAGTTTGTATTG	Cloning of pBAD24-*clbQ*-*rrnB*t
4	GCGCTCTAGAGCCATATCTATTGCTCCTTG	Cloning of pBAD24-*clbQ*-*rrnB*t
5	CCATCGAATGGCCAGATGAT	Cloning of pBAD24-*tetA*p-*clbQ*-*rrnB*t
6	GCGCACATGTAGCATTTATCAGGGTTATTGTCT	Cloning of pBAD24-*tetA*p-*clbQ*-*rrnB*t
7	TGGAAAAACGCCAGCAACG	Cloning of pBAD24-*tetA*p-*clbQ*-*rrnB*t
8	TTCACTTTTCTCTATCACTGATAG	Cloning of pBAD24-*tetA*p-*clbQ*-*rrnB*t
9	GTGATAGAGAAAAGTGAAATGAGTAATATCAGTTTGTATTGTTTG	Cloning of pBAD24-*tetA*p-*clbQ*-*rrnB*t
10	TGCTCATGGTAGTTACTCCTTTTC	Cloning of *clbR* deletion
11	CAATCCTCATGTTTCCCCCCATCCTGAATGGTATC	Cloning of *clbR* deletion
12	GGGGGGAAACATGAGGATTGATATATTAATTGGAC	Cloning of *clbR* deletion
13	CGCCATTTAAATGAAGTTCCTATTCCTCAATTCTGCCCATTTGACG	Cloning of *clbR* deletion
14	GCGCCACGTCGTACGTGCAAATATGGCAAACC	Cloning of *clbR* deletion
15	GTATAGGAACTTCTCAATTCTGCCCATTTGACG	Cloning of *clbR* deletion
16	GGGCAGAATTGAGAAGTTCCTATACTTTCTAGAGAATAG	Cloning of *clbR* deletion
17	CCTGTCTCTTGATCAGATCTTGATC	Cloning of *clbR* deletion
18	GGTGGTTGCTCTTCCGCAGATAATCTCATTCCTGTTAGC	Cloning of *clbR* deletion/ pTXB1_clbR
19	GGTGGTCATATGGATAAGTTCAAAGAAAAAAACC	Cloning of pTXB1_clbR
20	TGTTTCTCCATACCCGTTTTTTTGGGCTAGCAGGAGGAATTCACCATGGATAAGTTCAAAGAAAAAAACC	Amplification of *clbR* for cloning into pBAD24_clbR
21	TTCTGTTTTATCAGACCGCTTCTGCGTTCTGATTTAATCTGTATCATTAGATAATCTCATTCCTGTTAGC	Amplification of *clbR* for cloning into pBAD24_clbR
22	GCTAACAGGAATGAGATTATCTAATGATACAGATTAAATCAGAACGC	Amplification of *rrnB*t for cloning into pBAD24_clbR
23	CAAAAGAGTTTGTAGAAACGC	Amplification of *rrnB*t for cloning into pBAD24_clbR
24	TTTCGCCACCTCTGACTT	Amplification of *tetA*p for cloning into pGEM®-T Easy-tetAp-clbR
25	GGTTTTTTTCTTTGAACTTATCCATTTCACTTTTCTCTATCACTGATAGG	Amplification of *tetA*p for cloning into pGEM®-T Easy-tetAp-clbR
26	ATGGATAAGTTCAAAGAAAAAAACC	Amplification of *clbR*-*rrnB*t from pBAD24_clbR
27	AGCATTTATCAGGGTTATTGTCT	Amplification of *clbR*-*rrnB*t from pBAD24_clbR
28	CCATCGAATGGCCAGATGAT	Fusion PCR to fuse the *tetA*p and *clbR*-*rrnB*t amplicons

Primer	Sequence (5`-3`)	Function
29	CAAAAGAGTTTGTAGAAACGC	Fusion PCR to fuse the *tetA*p and *clbR*-*rrnB*t amplicons
30	AATGCCATCTGGTATCACTTAAAGGTATTAAAAACAACTTTTTGTCTTTTTACCTCATATGAATATCCTCCTTAGTTCC	Cloning of M1/5 *rpsL*K42R λ*attB*::FRT
31	AAGCCAATGCCAGCGCCAGACGGGAAACTGAAAATGTGTTCACAGGTTGCTCCGGGCTATGTGTAGGCTGGAGCTGCTTC	Cloning of M1/5 *rpsL*K42R λ*attB*::FRT
32	TTCCCGTGATGGATAAATAAG	Amplification *frr* promotor region
33	GTTACGAATCCTTGAAAACTT	Amplification *frr* promotor region
34	TATGCAGCAGCGACATAAGG	Fusing *luxA* with *frr* prmotor region
35	AAGTTTTCAAGGATTCGTAACATGAAATTTGGAAACTTTTTGCTTACATACCAAC	Fusing *luxA* with *frr* prmotor region
36	GACGGAAGATCACTTCGCAG	Amplification of *cat* cassette
37	ACACCAGGATTTATTTATTCTGCGAAGTGATCTTCCGTCTTCCCGTGATGGATAAATAAG	Fusing *luxA* overlap with *frr* promotor region, adding *cat* cassette overlap
38	GAGATAACGGGTTTTTTTCTTTGAACTTATCCATGTTTCCCCCCATCCTGTTACGCCCCGCCCTGCCACT	Scarless VNTR cloning
39	GCCTGTAGCAGTTGAGGAATTATAGCATCAATACCGTCATCCTGCGGGACGCTAGCTTCTTCGTCTGTTT	Scarless VNTR cloning
40	GAGATAACGGGTTTTTTTCTTTGAACTTAT	Scarless VNTR cloning
41	GCCTGTAGCAGTTGAGGAATTATAG	Scarless VNTR cloning
42	ACGAGTATCGAGATGGCACA	Control PCR for chromosomal *clbR* deletion
43	ATGGCTCTTGTATCTATC	Control PCR for chromosomal *clbR* deletion
44	GATAAGTTCAAAGAAAAAAACCCGTTATCTCTGCGTGAAAGACAAGTATTGCGCATGCTGTGTAGGCTGGAGCTGCTTC	Chromosomal *clbR* deletion
45	ATATGAAAATCAATATTATCGACGGCTCAGAAGTGTCTAGATTATCCGTGGCGATCATATGAATATCCTCCTTAGTTCC	Chromosomal *clbR* deletion
46	ACCGTTGTATCGTAAATTCCTC	Chromosomal *clbR* deletion
47	GATTGTATTGAATTTCATTTAAC	Chromosomal *clbR* deletion
48	TGCTCATGGTAGTTACTCCTTTTC	General primer probe generation for EMSA with *clbB* upstream region
49	CATCCTGAATGGTATCTGTGTATC	547-bp probe generation for EMSA with *clbB* upstream region (probe 1)
50	ATCTGTGTATCTGTTGTTTTGGCAG	478-bp probe generation for EMSA with *clbB* upstream region (probe 2)
51	TTATGTAAATGGGAATTACGC	422-bp probe generation for EMSA with *clbB* upstream region (probe 3)
52	TGTATGTACTGCATGACTTACATG	355-bp probe generation for EMSA with *clbB* upstream region (probe 4)
53	AACATGTAATCAGAATATAAGG	294-bp probe generation for EMSA with *clbB* upstream region (probe 5)
54	CAATATGATTATATGAATACGC	238-bp probe generation for EMSA with *clbB* upstream region (probe 6)
55	ATGTTAAATAATCTATAAATCC	171-bp probe generation for EMSA with *clbB* upstream region (probe 7)

Primer	Sequence (5`-3`)	Function
56	GAGATTTTCCATGTAATAAGATGG	115-bp probe generation for EMSA with *clbB* upstream region (probe 8)
57	TGAATACGCAATAAAAAACTATACTTG	225-bp probe generation for EMSA with *clbB* upstream region (probe 9)
58	AAAAACTATACTTGCGGATAGGTG	212-bp probe generation for EMSA with *clbB* upstream region (probe 10)
59	TGCGGATAGGTGTGCCATTTAG	200-bp probe generation for EMSA with *clbB* upstream region (probe 11)
60	CCATTTAGAATAATCATGTTAAATAATC	186-bp probe generation for EMSA with *clbB* upstream region (probe 12)
61	ACCGTTGTATCGTAAATTCCTC	General primer probe generation for EMSA with *clbR* upstream region
62	CTTTCACGCAGAGATAACGGG	176-bp probe generation for EMSA with *clbR* upstream region (probe 13)
63	GGTTTTTTTCTTTGAACTTATC	157-bp probe generation for EMSA with *clbR* upstream region (probe 14)
64	CATGTTTCCCCCCATCCTG	135-bp probe generation for EMSA with *clbR* upstream region (probe 15)
65	CATCCTGAATGGTATCTGTGTATC	123-bp probe generation for EMSA with *clbR* upstream region (probe 16)
66	CCGATTCATTAATGCAGCTG	229–bp probe generation for EMSA *lacZ* control
67	CCAGTCACGACGTTGTAATACG	229–bp probe generation for EMSA *lacZ* control
68	GTTGTCGCTGAAGCAACTGG	RT-qPCR *gapA*
69	AGCGTTGGAAACGATGTCCT	RT-qPCR *gapA*
70	CGTGGTGATAAAGTTGGGAC	RT-qPCR *clbA*
71	CTCCACAGGAAGCTACTAAC	RT-qPCR *clbA*
72	AGCGTGATTCGTATTCCGAG	RT-qPCR *clbR*
73	CCGTTATCTCTGCGTGAAAG	RT-qPCR *clbR*
74	CGACGTCACACCTTTCAGCACG	RT-qPCR *clbB*
75	TGCGGGGGTGATACGATAATCG	RT-qPCR *clbB*
76	ATCGTCAAGCTGGGCGGAACTC	RT-qPCR *clbQ*
77	AGTCCGCGTCTTTCGTGTGGTG	RT-qPCR *clbQ*
